# Confidence and second-order errors in cortical circuits

**DOI:** 10.1093/pnasnexus/pgae404

**Published:** 2024-09-13

**Authors:** Arno Granier, Mihai A Petrovici, Walter Senn, Katharina A Wilmes

**Affiliations:** Department of Physiology, University of Bern, Bühlplatz 5, Bern 3012, Switzerland; Graduate School for Cellular and Biomedical Sciences, University of Bern, Bern, Switzerland; Department of Physiology, University of Bern, Bühlplatz 5, Bern 3012, Switzerland; Department of Physiology, University of Bern, Bühlplatz 5, Bern 3012, Switzerland; Department of Physiology, University of Bern, Bühlplatz 5, Bern 3012, Switzerland

**Keywords:** cortical computation, predictive coding, uncertainty, energy-based models

## Abstract

Minimization of cortical prediction errors has been considered a key computational goal of the cerebral cortex underlying perception, action, and learning. However, it is still unclear how the cortex should form and use information about uncertainty in this process. Here, we formally derive neural dynamics that minimize prediction errors under the assumption that cortical areas must not only predict the activity in other areas and sensory streams but also jointly project their confidence (inverse expected uncertainty) in their predictions. In the resulting neuronal dynamics, the integration of bottom-up and top-down cortical streams is dynamically modulated based on confidence in accordance with the Bayesian principle. Moreover, the theory predicts the existence of cortical second-order errors, comparing confidence and actual performance. These errors are propagated through the cortical hierarchy alongside classical prediction errors and are used to learn the weights of synapses responsible for formulating confidence. We propose a detailed mapping of the theory to cortical circuitry, discuss entailed functional interpretations, and provide potential directions for experimental work.

Significance Statement Two prevailing notions in modern computational neuroscience have been that cortical processing follows principles of predictive coding and takes uncertainty into account. In this work, we propose a natural extension of predictive coding where cortical areas not only predict the activity in other areas but also formulate, exchange, and adjust their confidence (inverse expected uncertainty) about these predictions. This leads to a normative account of top-down gain modulation in cortical circuits and predicts the existence of a new class of cortical errors.

## Introduction

Taking uncertainty into account in models of cortical processing has proven beneficial to capture behavioral and neural data at multiple scales (1–3). Empirical studies on humans and other animals show that prior knowledge and data from multiple modalities are weighted by their relative uncertainty during perceptual integration (4, 5), decision-making (6, 7), and sensorimotor control (8, 9). Crucially, uncertainty is context-dependent and can vary dynamically (10, 11). For example, in the dark, animals should rely more on prior knowledge of the environment than vision, whereas in daylight, they can trust their vision more.

Additionally, cortical processing has been described based on the notion of prediction (12, 13), with cortical areas attempting to predict the activity in other areas or sensory streams. The computational goal of the cortex would then be to minimize differences between these predictions and actual activity, commonly referred to as prediction errors. Neural computations realizing this goal have been proposed as canonical cortical computations (14–16). One way to incorporate uncertainty in these models is to assume that a cortical prediction should not simply be a single potential representation of the target area but rather a distribution over the space of possible representations. In that case, normative theories based on variants of maximum-likelihood estimation suggest that cortical prediction errors should be multiplicatively weighted by the inverse variance of the predictive distribution. This modulatory weighting of prediction errors has gained a central place in the branch of cognitive sciences based on predictive coding (17, 18), most notably in models of attention (19–21), and in neuropsychiatry (22–25). Potential neural implementations have been discussed, notably in cortico-pulvinar loops including populations of neurons encoding beliefs about uncertainty (17, 26) (also see ref. (27) for a purely cortical implementation), or more generally through neuromodulation (28, 29). However, a formal account of the role of learned and context-dependent uncertainty estimation is still missing.

In this work, we suppose that cortical areas must not only predict the activity in other areas and sensory streams but also jointly estimate the confidence of their predictions, where we define confidence as the (estimated) inverse expected uncertainty of the prediction. In other words, we introduce measures of confidence computed at each level of the cortical hierarchy as a function of current higher-level representations, forming a hierarchy of confidence analogous to the hierarchy of predictions. For example, the representation of the environment will determine the degree of confidence in a prediction about the presence of a particular object (e.g. this confidence will be different in familiar versus unfamiliar environments). Similarly, the representation of an object will determine the degree of confidence in predictions of lower-level features of that object (e.g. some objects are always of the same color while others can vary). This formulation is in line with rare instances where inverse uncertainty has been formulated as a function of current neuronal activity (19, 26), and to be contrasted with the majority of literature in which it is predominantly defined as a parameter of the internal model, independent of current neuronal activity. With our formulation, confidence has a fast, dynamic, and context-dependent influence on neural dynamics, while the parameters of the function computing confidence, encoded in synaptic weights, slowly learn the statistics of the environment. Our definition of confidence differs from the everyday use of the term reflecting metacognitive or subjective feelings of confidence. However, subjective confidence might emerge from probabilistic neural representations like the one we consider in this work (30). Moreover, it is interesting to note that experiments at the behavioral level recently confirmed that the brain forms estimates of metacognitive confidence based on prior knowledge (31).

## Results

### An energy for cortical function

Given the organization of the cortex into specialized areas, we define latent cortical representations as $u_{1},\ldots ,u_{n}$, corresponding to the membrane potentials of neuronal populations in *n* areas, and denote $u_{0}$ the observation. For example, the observation $u_{0}$ might be the activity of visual sensors (retina), and latent cortical representations $u_{1},\ldots ,u_{n}$ might encode local orientation (V1), color (V4), objects (IT), etc.

**Fig. 1.**
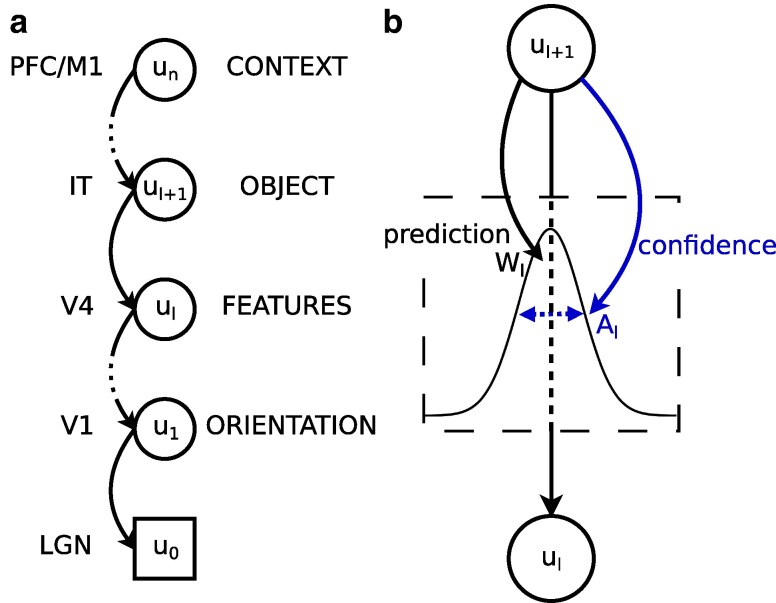
Predictive distributions in the cortical hierarchy. a) Probabilistic model. Latent representations ($u_{\ell }$) are organized in a strict generative hierarchy. b) Predictions are Gaussian distributions. Both the mean ($\mu_{\ell }=W_{\ell }r_{\ell +1}$, first-order) and the confidence ($\pi_{\ell }=A_{\ell }r_{\ell +1}$, inverse variance, second-order) are functions of higher-level activity.

As a simplifying assumption, we organize areas in a strict generative hierarchy, such that area ℓ+1 tries to predict the activity of only the area below (see Fig. 1a). It does so by sending its output rates $r_{\ell +1}=\phi (u_{\ell +1})$ through top-down synapses with plastic weights $W_{\ell }$, where *ϕ* represents the neuronal activation function. Additionally, area ℓ+ℓ similarly estimates and conveys to area the confidence of its prediction through top-down synapses with plastic weights $A_{\ell }$. We further hypothesize that the resulting predictive distribution is the (entropy-maximizing) normal distribution with mean vector $\mu_{\ell }=W_{\ell }r_{\ell +1}$ and confidence (inverse variance) vector $\pi_{\ell }=A_{\ell }r_{\ell +1}$ (see Fig. 1b). Crucially, confidence is not simply a static parameter of the model; instead, it is a parameterized function of current higher-level representations. For example, different context representations might lead to different levels of certainty about the presence of the same object, and different object representations might send more confident predictions for one sensory modality than another. In essence, this is an extension of the notion of prediction, where cortical areas predict the confidence (second-order information) in addition to the mean (first-order information).

We can now formulate our energy (or cost) for cortical function


(1) $$E=\frac{1}{2}\sum_{\ell =0}^{n-1}\|e_{\ell }{\|}_{\pi_{\ell }}^{2}-\frac{1}{2}\sum_{\ell =0}^{n-1}\log\;|\pi_{\ell }|,$$

where $e_{\ell }=u_{\ell }-\mu_{\ell }$ is a prediction error, $\|\cdot {\|}_{\pi_{\ell }}$ denotes the norm with $\pi_{\ell }$ as a metric (i.e. a variance-normalized norm, $\|e_{\ell }{\|}_{\pi_{\ell }}^{2}=e_{\ell }^{T}\text{diag}(\pi_{\ell })e_{\ell }$) and |⋅| denotes the product of components. This energy can be derived as the negative log-joint of a hierarchical generative probabilistic model (see Materials and methods). Note that $\|e_{\ell }{\|}_{\pi_{\ell }}$ is the classical Euclidean norm of standardized errors. In other words, here, we measure distances in terms of numbers of standard deviations away from the mean. This metric, the Mahalonobis distance, is a better measure of distance between a point (representation) and a Gaussian distribution (prediction) than simply the Euclidean distance to the mean $\left\|e_{\ell }\right\|$.

This energy *E* seems worth minimizing. The first term is a measure of distance between actual representations and predictions, additionally taking into account the confidence of predictions: the more a prediction is confident, the more a deviation from it matters. The second term indicates that high confidence is preferable. In other words, the cortex tries to reduce its expected uncertainty. That is, as long as high confidence does not excessively lead to an increase in the first term: there must be a balance between the confidence and the (average) magnitude of prediction errors (defined as the squared unsigned prediction errors). In other words, areas learn to be confident in predictions leading to small remaining errors ($e_{\ell -1}^{2}$). Moreover, the second term also acts as a regularizer to avoid uninformative, i.e. very small, confidence.

Having formulated an energy for cortical function, we formally derive gradient-based neuronal dynamics and synaptic learning rules minimizing this energy.

### Neuronal dynamics with confidence estimation

**Fig. 2.**
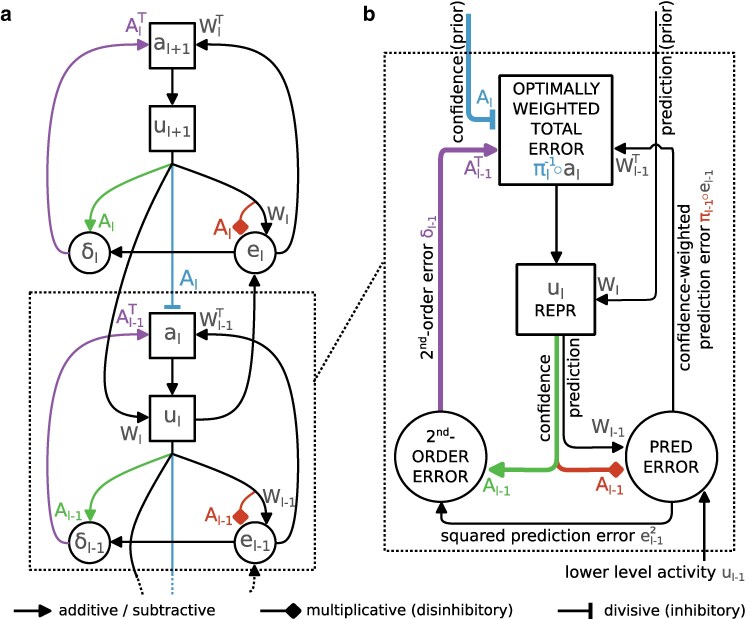
Neuronal dynamics of inference. a) A high-level schematic depiction of neuronal dynamics (Eqs. 2 and 3). Prediction errors [$e_{\ell }$] are first computed by comparing predictions [$\mu_{\ell }=W_{\ell }r_{\ell +1}$] with actual activity or data [$u_{\ell }$]. Prediction errors are weighted multiplicatively by the estimated confidence (inverse expected variance) of the prediction [$\pi_{\ell }=A_{\ell }r_{\ell +1}$]. The second-order errors [$\delta_{\ell }$] are computed by comparing inverse confidence estimates [$\pi_{\ell }^{-1}$] and squared prediction errors [$e_{\ell }^{2}$]. The second-order errors are up-propagated and integrated alongside up-propagated prediction errors into the total error [$a_{\ell }$]. The total error is divisively modulated by the prior confidence [$\pi_{\ell }$] b) A more detailed illustration centred on dynamics for representations at a single level ℓ. Prediction errors and second-order errors are then those of level ℓ−1.

We classically derive neuronal dynamics of inference minimizing the energy *E* through gradient descent. Moreover, we make use of confidence $\pi_{\ell }$ as a metric to guide our descent (32). The resulting dynamics can be interpreted as an approximate second-order optimization scheme (see Materials and methods). This leads to the leaky neuronal dynamics


(2) $$\tau \dot{u_{\ell }}=-\pi_{\ell }^{-1}\circ \partial E/\partial u_{\ell }=-u_{\ell }+\mu_{\ell }+\pi_{\ell }^{-1}\circ a_{\ell },$$

integrating top-down predictions $\mu_{\ell }=W_{\ell }r_{\ell +1}$, and total propagated errors


(3) $$a_{\ell }=r_{\ell }^{\prime }\circ (W_{\ell -1}^{T}(\pi_{\ell -1}\circ e_{\ell -1})+A_{\ell -1}^{T}\delta_{\ell -1})$$

defined as the sum of confidence-weighted prediction errors $\pi_{\ell -1}\circ e_{\ell -1}$ and second-order errors $\delta_{\ell -1}=(\pi_{\ell -1}^{-1}-e_{\ell -1}^{2})/2$, both propagated upwards from the lower area. Here ∘ is the componentwise (Hadamard) product and $e_{\ell -1}^{2}=e_{\ell -1}\circ e_{\ell -1}$. The dynamics entailed by Eqs. 2 and 3 are illustrated in Fig. 2. The second-order errors $\delta_{\ell }$ are not errors on the prediction (of the mean) $\mu_{\ell }$ but errors on the confidence $\pi_{\ell }$, which are expected to be on average 0 if and only if the estimate $\pi_{\ell }$ correctly captures the underlying inverse variance. Following previous work (33), we suppose that total propagated errors $a_{\ell }$ are encoded in the apical dendrites of cortical neurons with somatic membrane potential $u_{\ell }$.

**Fig. 3.**
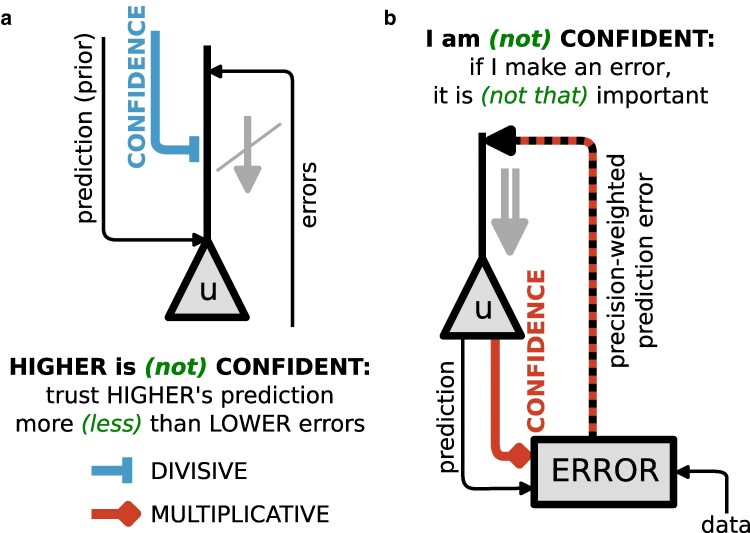
Adaptive balancing of cortical streams based on confidence. a) Divisive modulation of errors by the confidence of top-down predictions about what the activity of a neuron should be (prior confidence, $\pi_{\ell }^{-1}$). b) Multiplicative modulation of errors by the confidence of predictions that a neuron makes about what the activity of other neurons should be (data confidence, $\pi_{\ell -1}$).

**Fig. 4.**
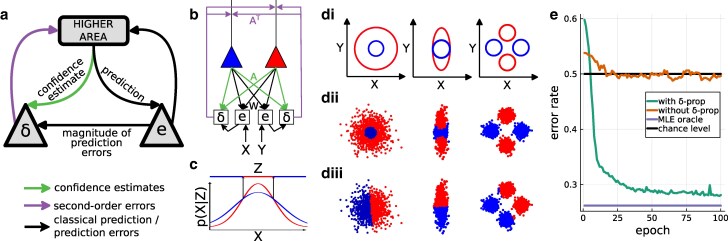
Propagation of second-order errors for classification. a) The second-order errors compare confidence and performance (with performance defined as a function of the magnitude of prediction errors). b) A 2×2 network for binary classification. During learning, the X and Y data are sampled from one of the two class distributions, and the activity of neurons representing the class is clamped to the one-hot encoded correct class. Parameters (W,A) are then learned following Eqs. 4 and 5. During inference, the activity of neurons representing the class follows neuronal dynamics (without top-down influence), and we read the selected class as the one corresponding to the most active neuron. Prediction error (first-order) propagation is omitted in the depiction. c) Maximizing the likelihood of predictions leads to nonlinear classification in a single area. di) Two different 2-dimensional binary classification tasks. The ellipse represents the true class distributions for the two classes. dii) Classification with second-order error propagation. diii) Classification without second-order error propagation. e) Classification accuracy on the task presented in d, second column.

These neuronal dynamics (Eqs. 2 and 3) entail two major points of interest, one of gain modulation of errors based on confidence (see Fig. 3) and one of second-order error propagation (see Fig. 4). Mechanisms of gain modulation can further be subdivided into a divisive modulation by prior confidence $\pi_{\ell }$, and a multiplicative modulation by data confidence $\pi_{\ell -1}$. In the following section, we complete our theoretical framework by deriving synaptic learning rules for parameters $W_{\ell }$ and $A_{\ell }$. We then return to neuronal dynamics and further unpack these two points of interest.

### Error-correcting synaptic learning of confidence

At the equilibrium of neuronal dynamics, weights of synapses carrying predictions can be learned following the gradient


(4) $$\dot{W_{\ell }}\propto -\partial E/\partial W_{\ell }=(\pi_{\ell }\circ e_{\ell })r_{\ell +1}^{T},$$

where $\pi_{\ell }\circ e_{\ell }$ are postsynaptic confidence-weighted prediction errors and $r_{\ell +1}$ are presynaptic rates. This is the classical learning rule for prediction weights in the predictive coding framework (14). By following this learning rule, synapses learn to correctly predict lower-level features (e.g. orientation) from higher-level activity (e.g. object). Additionally, confidence impacts learning speed: if a prediction is confident but wrong, a significant update is required, whereas an error on a prediction made with low confidence might reflect intrinsic variability and does not require a big update.

Similarly, weights $A_{\ell }$ of synapses carrying confidence can also be learned following the gradient


(5) $$\dot{A_{\ell }}\propto -\partial E/\partial A_{\ell }=\delta_{\ell }r_{\ell +1}^{T},$$

where again $\delta_{\ell }=(\pi_{\ell }^{-1}-e_{\ell }^{2})/2$ are postsynaptic second-order errors. By following this learning rule, synapses learn to correctly estimate the confidence of the associated prediction, which we use as a context-specific metric. Since $\pi_{\ell }=A_{\ell }r_{\ell +1}$ approximates an inverse variance and enters as a metric in Eqs. 1 and 2, it should remain positive. An important extension of Eq. 5 is then to include a mechanism to ensure that components of $A_{\ell }$ remain positive (see Materials and methods).

These two similar learning rules state that synaptic weights evolve to minimize errors remaining after inference. We verify in simulations that Eqs. 4 and 5 (with an additional mechanism to ensure positivity, see Materials and methods) can indeed learn correct mean and confidence of different context-dependent data distributions as functions of higher-level representations (see Supplementary Material, SI5). Importantly, all the information needed for learning, namely the presynaptic rate and postsynaptic error, is readily available in the vicinity of the synapse.

Having developed a way to learn how to estimate top-down confidence, we will now further examine how this is used in neuronal dynamics.

### Dynamic balancing of cortical streams based on confidence

In our neuronal dynamics (Eqs. 2 and 3), the relative importance given to top-down predictions and bottom-up prediction errors is controlled by two mechanisms that both modulate the gain of prediction errors. First, the confidence of top-down predictions of a neuron’s activity (“prior confidence”) divisively impacts the importance of bottom-up errors in the inference dynamics of this neuron (see Fig. 3a). For example, neurons encoding context might send more or less confident predictions to neurons encoding the presence of particular objects. Then, the relative importance of the prior prediction compared to bottom-up errors is greater in contexts sending more confident prior predictions (“In a forest, I know there are trees”) than less confident ones (“In this city neighbourhood, there might be trees, let’s see…”).

Second, the confidence of predictions a neuron makes about lower-level activities (“data confidence”) multiplicatively impacts the importance of errors entailed by these predictions (see Fig. 3b). For example, neurons encoding object identity might send predictions to different sensory modalities with different confidence levels, reflecting different levels of reliability or noise in different lower-level streams. Prediction errors arising from more reliable streams should be weighted more strongly (“Across trees, structure (trunk, branches, leaves, etc.) is usually more consistent than color. To recognize a tree, I should then trust structure more than color”).

This weighting is proportional to the more classical Bayes-optimal weighting of top-down prediction (akin to prior) and bottom-up errors (akin to data) by their respective reliabilities, and leads to a Bayes-optimal estimate of latent variables at equilibrium of neuronal dynamics. Computationally, this mechanism proves valuable when integrating information from sources with different levels of reliability (or noise), for example, when integrating prior and data or during multimodal integration (see Supplementary Material, Fig. S4 and SI6).

At the level of a cortical area, confidence controls the balance of bottom-up and top-down information on a neuron-by-neuron basis, providing fine-grained control over what is attended to. It is worth highlighting that, with our formulation of confidence as a function of higher-level representations, we can encompass state-, context-, task-, or feature-dependent confidence signals, depending on what the higher-level representations encode. Moreover, as higher-level representations change, so do confidence signals, providing a mechanism to explain the trial-to-trial variability of confidence weighting observed in animals (10) (see Fig. S4).

### Second-order error propagation

In the proposed neuronal dynamics (Eqs. 2 and 3), second-order errors $\delta_{\ell }$ are propagated through the cortical hierarchy alongside confidence-weighted prediction errors $\pi_{\ell }\circ e_{\ell }$ (see Fig. 4a). This entails a second-order cortical stream along which areas exchange confidence and second-order errors. Importantly, this means that the second-order errors change higher-level representations (see Fig. S4).

To investigate the computational role of second-order error propagation and their influence on higher-level representations, we place a single area (a network without hidden layers, see Fig. 4b) in supervised learning settings on simple nonlinear binary classification tasks (see Fig 4di and Materials and methods). Parameters are learned following Eqs. 4 and 5. As expected, the confidence signal after learning represents the class-specific inverse variance (see Fig. S1). With our dynamics (see Fig. 4dii), but not with classical predictive coding dynamics (see Fig. 4diii), a single area can solve these nonlinear classification tasks (see Fig. 4e).

At a computational level, this qualitative difference in performance (classification accuracy) can be understood by looking at the energy we minimize. With our model, we choose the latent representation which sends a predictive distribution with the highest likelihood with respect to current data (see Fig. 4c). In contrast, classical predictive coding chooses the latent representation that minimizes the Euclidean distance between the input and the entailed point prediction. At an algorithmic level, the capacity of our network to solve these tasks comes from the influence of second-order errors on the higher-level representation. To minimize second-order errors, the network must not only choose the class whose point (mean) prediction is closest to the data point (that is, first-order prediction error minimization). This is noninformative in the example in Fig. 4 because both class distributions have the same mean. The network also has to choose the class that best predicts the remaining distance ($e_{\ell }^{2}$) between point prediction and data.

### Confidence estimation in cortical circuits

**Fig. 5.**
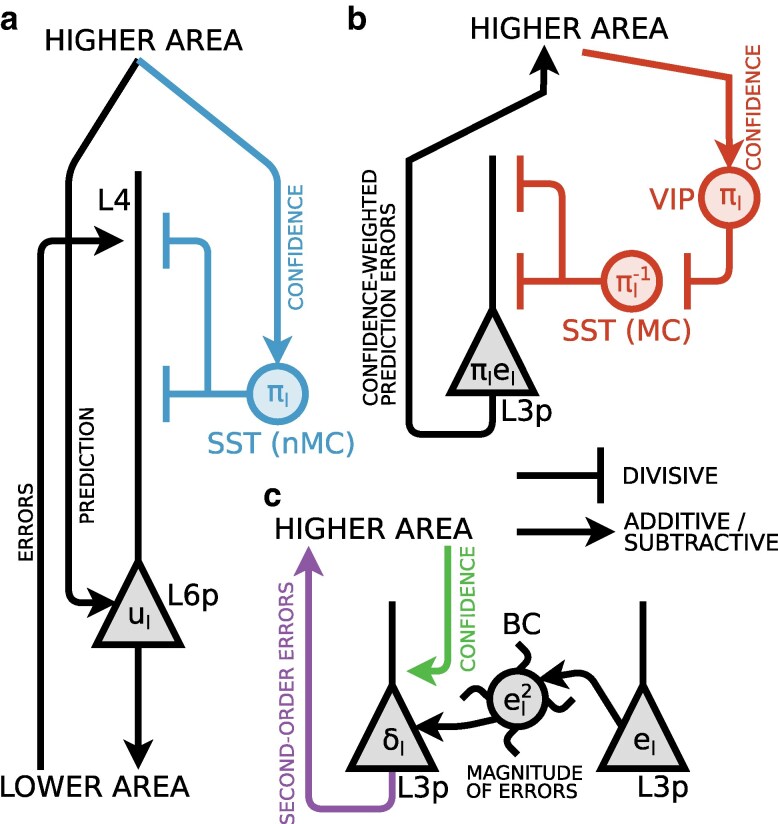
Cortical circuit for neuronal dynamics of inference (as described in Eq. 2 and Eq. 3). a) Representations ($u_{\ell }$) are held in the somatic membrane potential of L6p. Top-down synapses carrying predictions ($\mu_{\ell }=W_{\ell }r_{\ell +1}$) directly excite L6p at proximal dendrites. Bottom-up confidence-weighted prediction errors ($W_{\ell -1}^{T}(\pi_{\ell -1}\circ e_{\ell -1})$) and second-order errors ($A_{\ell -1}^{T}\delta_{\ell -1}$) are integrated into total error ($a_{\ell }$) in the distal dendrites of L6p as described in Eq. 3. This total error is then weighted by the prior uncertainty ($\pi_{\ell }^{-1}$) through divisive dendritic inhibition realized by deep SST-expressing interneurons. b) Top-down predictions ($\mu_{\ell }=W_{\ell }r_{\ell +1}$) and local representations ($u_{\ell }$) are compared in L3*e*. Confidence weighting is then realized through gain modulation of L3*e* by the disinhibitory VIP-expressing and SST-expressing interneurons circuit. c) L3*δ* compares top-down confidence and local squared prediction errors encoded in basket cells (BC) into re-weighted second-order errors.

We next describe how our dynamics could be realized in cortical circuits (see Fig. 5; for an illustration of the entire cortical ensemble, see Fig. S3). We postulate that latent variables $u_{\ell }$ are encoded in the somatic activity of a population of intracortical pyramidal cells of layer 6 (L6p). As demanded by our theoretical framework, these neurons receive the majority of their input from intracortical long-range projections (34) and send top-down projections to lower cortical areas (35, 36). We propose that these projections carry not only predictions (37–39), but also confidence. Following experimental evidence of error or mismatch encoding in pyramidal cells of cortical layer 2/3 (40, 41), we propose that confidence-weighted prediction errors $\pi_{\ell }\circ e_{\ell }$ and second-order errors $\delta_{\ell }$ are computed by two populations of pyramidal neurons situated in layer 3, respectively L3*e* and L3*δ*. As our theory demands, these neurons send feedforward projections to higher cortical areas (35, 36). Additionally, our theory suggests that both types of error are integrated into the total propagated errors $a_{\ell }$ (as defined in Eq. 3). We propose that this integration takes place in distal apical dendrites of L6p situated at the height of layer 4/5a (42), in line with previous work postulating error encoding in segregated dendritic compartments (33).

We now concern ourselves with the balancing of cortical streams through inhibition and disinhibition of errors entailed by our theory. We propose that prediction errors are computed in L3*e* by comparing local and top-down inputs from L6p. The weighting of bottom-up prediction errors by (data-) confidence might be realized through top-down gain modulation targeting L3*e*. This could be achieved through a well-known disinhibitory circuit motif involving Vasoactive intestinal peptide (VIP)-expressing interneurons receiving top-down input and preferentially inhibiting Somatostatin (SST)-expressing interneurons which in turn preferentially inhibit dendrites of L3*e* (43–45) (see Fig. 5b). This would entail VIPs encoding a confidence signal and superficial SSTs an expected uncertainty signal. This hypothesis is corroborated by recent 2-photon imaging on rodents placed in an oddball paradigm, where activity ramps up in VIPs and decays in superficial SSTs as a stimulus is repeated (46). Moreover, our theory suggests that total bottom-up errors should be modulated by the (prior-) uncertainty of top-down predictions (the factor $\pi_{\ell }^{-1}$ in Eq. 2). In other words, at a circuit level, the confidence in prior, top-down information controls the integration of bottom-up errors by modulating the gain of somatic integration of apical activity. We propose that this is realized through modulation of L6p apical dendrites by deep (non-Martinotti) SST interneurons, which would then encode a confidence signal (see Fig. 5a). The laminar specificity of SST activity (47) and targets (48) supports this hypothesis.

Finally, we suggest circuit-level mechanisms underlying second-order error computation in L3*δ* (see Fig. 5c). We caution here that the schematic of Fig. 5c should not be taken too literally as circuit mechanisms, and rather illustrates the key concept that to compute second-order errors, confidence must be compared to the magnitude of current prediction errors. We propose that the magnitude of prediction errors is computed in Parvalbumin (PV)-expressing basket cells from local L3*e* inputs. At a circuit level, L3*e* is thought to be separated into two populations encoding the positive and negative part of prediction errors, respectively (41). If this holds, then excitatory projections from both these populations to local basket cells, eventually followed by a nonlinear integration by basket cells (49), would be sufficient to perform the needed computation of local error magnitude (50). L3*δ* would then compute second-order errors by comparing top-down confidence and local (subtractive) inputs from basket cells. PV-expressing basket cells have indeed been shown to preferentially inhibit specific pyramidal cell types (51, 52). The here presented propositions can serve as a starting point for experimental investigation of cortical second-order errors.

The presented mapping to cortical circuits allows us to make the following experimental predictions, beyond those made by more classical predictive coding models:

1. Feedback connections originating from deep layer pyramidal cells (in our case, L6p) carry predictions or confidence estimates depending on the postsynaptic target cell type. In other words, both predictions and confidence estimates are functions of the activity of higher-level deep pyramidal cells.
2. Neural signatures of confidence estimates can be found in VIP and SOM interneurons. The activity of these interneurons should notably be controlled by higher-level representations.
3. The activity of supragranular and infragranular SOM interneurons inversely covary.
4. The strength of apical modulation, particularly targeting the apical integration zone, is proportional to the top-down expected uncertainty.
5. Basket cells encode the magnitude of local prediction errors, potentially by integrating the activity of positive and negative prediction error neurons.
6. One class of layer 2/3 pyramidal cells encodes second-order errors, comparing top-down confidence and actual predictive performance. Recent evidence suggests that L3*e* expresses *Adamts2* and *Rrad* (53), while no functional role has yet been proposed for the third class of superficial pyramidal cells expressing *Agmat*, which we propose could be L3*δ*.
7. Layer 2/3 pyramidal cells encoding errors project feedforward in the cortical hierarchy. This allows us to putatively more precisely situate them in deeper layer 3 (35, 36). These feedforward projections target the apical dendrites of deep pyramidal cells in the upper area, situated in layer 4/5a (considering layer 6 pyramidal cells).
8. Data confidence weighting. In an experiment where a contextual cue indicates which of two sensory modalities (e.g. vision and touch) is relevant to form a decision, we expect prediction error responses to be of greater magnitude for the relevant modality (see Supplementary material, Fig. S4a–e).
9. Prior confidence weighting. In an experiment where a contextual cue indicates directly which decision is correct and that sensory input should be ignored for this trial, we expect a strong inhibition of the apical integration zone of pyramidal cells encoding the decision variable, suppressing the integration of bottom-up sensory errors (see Supplementary material, Fig. S4a,b,f,g).
10. Second-order errors. In an experiment where the subject has to infer context based on the variability of sensory inputs rather than on the mean value (e.g. Context 1: [38,40,42]°; Context 2: [10,40,70]°), then we expect second-order errors to occur and drive inference (see Supplementary material, Fig. S4h,i).

## Discussion

In this work, we derived predictive coding dynamics with adaptive, context-dependent, and learned confidence information. Specifically, we considered diagonal estimates of the inverse covariance matrix (with diagonal $\pi_{\ell }$). In that case, each input dimension is scaled by the corresponding standard deviation when computing distances. However, the brain’s utilization of (inverse) variance estimates is likely to encompass various forms beyond the diagonal estimates explored in our study. Scalar estimates would define the importance granted to all errors in an area. In that case, confidence weighting of errors might be realized through nonspecific release of neuromodulators (28, 29), scaling all feature dimensions equally. A prime example of this could be found in the estimation of environmental variability and its use in perceptual decision-making (29). Taking this reduction to the extreme, the brain might make use of a single, global scalar confidence estimate. On the other end of the spectrum, we might consider full inverse covariance matrices. We would then consider not only stretch but also skew in our metric. Doing so might lead to a theoretically grounded account of lateral connections between prediction error nodes (15), with links to the notion of statistical whitening (the matrix square root of the full inverse covariance matrix is the ZCA whitening matrix). In general, we emphasize that considering the variance of predictive distributions as the backbone for normative theories of cortical modulation seems to us a promising endeavor.

Moreover, we treated predictions (mean estimates) as arising in a top-down manner, supposing that the cortex performs inference and learning on a purely generative model of its inputs. Considering bottom-up instead of top-down predictions is mathematically straightforward, could potentially align better with models considering top-down cortical pathways as modulating activity in a feedforward feature detection stream (54, 55), and could facilitate more direct testing on discriminative machine learning benchmarks. At the level of cortical circuitry, one might consider forward-projecting layer 5a and backward-projecting layer 2/3a pyramidal cells as sending bottom-up predictions and the entailed top-down prediction errors, respectively. This would form a fundamentally discriminative cortical pathway, a sort of dual of the generative pathway considered in this work.

It is important to acknowledge that the dynamics that we presented in this work share some classical limitations of predictive coding dynamics concerning biological plausibility e.g. weight transport (56), long inference (57), encoding of signed errors (33, 41), one-to-one connections, weak criteria of locality for learning and the assumption of a strict hierarchy of latent variables (58). Notably, our proposed circuitry necessitates that each L3e is paired with exactly two interneurons, and each L6p with one interneuron and one L3e, and that these are connected through one-to-one connections. This poses a limitation to the biological plausibility of the model, and future work might consider extensions relaxing those constraints developed in related literature (33, 59). Moreover, relaxing the simplifying hypothesis of a strict hierarchy of areas towards a model where “feedback” connections from different cortical areas participate in the confidence estimate in a single target area would help capture confidence estimates taking into account both sensory and decision confidence (7, 30, 60). Finally, a limitation at the circuit level is that second-order errors would need to be communicated to top-down synapses targeting VIP and infragranular SST interneurons to realize the learning rule Eq. 5.

In our model, confidence multiplicatively modulates errors and is computed at each level of the hierarchy as a function of current representations. This dynamic gain modulation is reminiscent of the attentional mechanism in transformer networks (61). Our formulation offers a first step towards a bridge between models of attention in terms of neural gain modulation based on confidence (19–21) and attentional mechanisms in machine learning. Anecdotally, VIP-expressing interneurons, encoding confidence in our model, were described by experimentalists as “generating a spotlight of attention” (62). Furthermore, the computational interest of dynamic top-down gain modulation might also be sought through the lens of efficient and parsimonious coding (63). This perspective may already be implicitly embedded within our framework, given the connection between maximum likelihood and the infomax principle (64).

A possible interpretation of the quantity encoded in second-order errors is as a form of “unexpected” uncertainty, as the difference between confidence estimates (“expected” uncertainty) and actual deviation from predictions. In that sense, second-order errors might be considered signatures of surprise, if we define surprise as unexpected predictive power (either better or worse than expected). In general, there is a tension between confidence-weighting and surprise-weighting in models of cortical computation, as encapsulated in the “perceptual prediction paradox” (65). Our model speaks to the resolution of this paradox also proposed in ref. (65): instantaneous and continuously computed confidence estimates initially bias perception (at level ℓ), while second-order propagation (to level ℓ+1) informs and refines inference subsequently, highlighting “surprising” events.

Confidence weighting of prediction errors occurs as a central element in leading models of psychopathologies under the predictive processing framework (22–25). These models are often based on the idea of a pathological (over- or under-) weighting of either prior or data in a process of Bayesian integration. In our model, these two hypotheses involve distinct neural mechanisms, that is, modulation, respectively, of L6p apical dendrites and L3p. This distinction might prove critical to extending these models to the whole cortical hierarchy, where activity at one level both represents data for the level above and generates priors for the level below. Moreover, our proposed computational roles for interneuron circuitry might help link accounts of neuropsychiatric disorders in terms of confidence weighting of errors to accounts in terms of cortical excitation–inhibition balance (66) and interneuron dysfunction (67).

On a similar note, a large body of experimental literature has focused on disentangling the neural signatures of expectation, prediction, and attention, often interacting in complex ways (e.g. (11, 68–71)). In this work, we introduce a formal distinction between prediction as the mean of a predictive distribution, and attention as the (inverse) variance of the same predictive distribution (equivalently: attention as a metric on the error landscape). This distinction entails different circuit mechanisms underlying prediction (L6p→L6p, L6p→L3e) and attention (L6p→VIP→L23-SOM→L3e disinhibition, L56-SOM→L6p apical dendrite inhibition). We hope that this formal distinction will help disentangle prediction and attention both in modeling and in more precise experiments targeting specific cell-types or subcircuits.

It has previously been suggested that prediction error responses of layer 2/3 cells should be modulated by the expected uncertainty of the predicted feature (72). Our derivation suggests that the same prediction errors should in addition be weighted by the expected uncertainty of the feature generating the prediction. Accordingly, we propose the terminology of doubly uncertainty-modulated prediction errors.

Finally, the suggested implementation in the circuitry of cortical pyramidal cells and interneurons definitely requires further refinement through experimental work. Nevertheless, we provide a rigorous theoretical framework to interpret existing experimental results and formulate ideas for experimental testing. Beyond providing a specific set of predictions, we aim to convey a novel normative perspective which indicates that searching for signatures of confidence estimation and second-order errors in cortical circuits might be an interesting venture, especially in interneuron activity.

## Materials and methods

### Probabilistic model

Here we elaborate on the form of the probabilistic model. We introduce a notion of strict hierarchy between levels of latent representations by supposing that the joint can be decomposed as


(6) $$p(u_{0},u_{1},\ldots ,u_{n})\propto p(u_{0}\;|\;u_{1})p(u_{1}\;|\;u_{2})\ldots p(u_{n-1}\;|\;u_{n}),$$

which can be justified by assuming a Markov property$\forall \;\ell \in [0,n),p(u_{\ell }\;|u_{\ell +1},\ldots ,u_{n})=p(u_{\ell }\;|\;u_{\ell +1})$ and a uniform top level prior $u_{n}∼U$. Since the distribution of $u_{\ell }$ is conditioned on $u_{\ell +1}$, we call this a generative hierarchy.

We further assume that the density of predictive distributions $p(u_{\ell }\;|\;u_{\ell +1})$ is multivariate Gaussian,


(7) $$p(u_{\ell }\;|\;u_{\ell +1})=f\left(u_{\ell };W_{\ell }r_{\ell +1},\text{diag}(A_{\ell }r_{\ell +1}{)}^{-1}\right)$$

where *f* is the multivariate Gaussian density with mean at point predictions $W_{\ell }r_{\ell +1}$ and diagonal covariance matrix with diagonal $(A_{\ell }r_{\ell +1}{)}^{-1}$.

Under the two assumptions described in Eqs. 6 and 7, we have


(8) $$-\log\;p(u_{0},u_{1},\ldots ,u_{n})+\mathrm{const}=E.$$

Despite this origin of our energy in probability, it is useful here to caution that our network entails only parametric representations of the distributions defined in Eq. 7. Moreover, never in our neuronal dynamics do we sample from these distributions, or in fact any distribution. Rather, our neuronal dynamics Eq. 2 refine representations $u_{\ell }$ towards a good (maximum a posteriori) point estimate of latent variables. This is to be contrasted with e.g. recent work that aim to sample from the posterior distribution in predictive coding networks (73).

### Confidence as metrics in neuronal dynamics

In this work, we chose confidence as a metric for neuronal dynamics Eq. 2 (see (32) for an introduction to the use of metrics in gradient-based dynamics in neuroscience). Note that if we make the approximation of considering that predictions are fixed during inference (a “fixed-prediction assumption” (74)), the confidence is the second derivative of the energy. Second derivatives provide additional information on the curvature of the energy landscape and are known to have desirable properties as metrics (second-order optimization). A striking limitation, however, lies in assuming fixed predictions during inference, the confidence is only a crude approximation of the actual second derivative without fixed predictions (see Supplementary Material, SI2 for the actual second derivative). An intuition of the effect of this change of metric on neuronal dynamics (Eq. 2) is as normalizing the balance of importance between local and lower prediction errors, such that the importance of local errors is 1.

### Positivity of confidence

A sufficient condition for neuronal dynamics Eq. 2 to follow a descent direction on *E* is that all terms of $\pi_{\ell }=A_{\ell }r_{\ell +1}$ are positive. Let us assume that rates $r_{\ell +1}$ are positive (the neuronal transfer function *ϕ* outputs positive values). Then a sufficient condition is that all components of $A_{\ell }$ also are positive. There are multiple possible extensions of Eq. 5 to enforce this. One is to initialize all components of $A_{\ell }$ to positive values and to modulate the learning rate by the current weights


(9) $$\dot{A_{\ell }}\propto A_{\ell }\circ \delta_{\ell }r_{\ell +1}^{T},$$

essentially preventing weights from crossing 0. This is necessary to stabilize learning when scaling up to more complex settings (see Supplementary Material, SI5). This is also in accordance with the general physiological fact that the sign of synaptic influence cannot change.

### Simulation details

For the simulations presented in Fig. 4, we built the datasets by sampling N=1000 points $\left(x_{1},y_{1}),\ldots ,(x_{N},y_{N}\right)$ from each of the data distributions represented in Fig. 4di (by their 99.7% confidence ellipses), and attaching the corresponding class label (either red or blue). We then build a 2x2 network where the top level activity is a one-hot representation of the class label and the bottom level activity is the coordinate in space (x,y). We train this network in supervised learning settings on the dataset by clamping both the top and bottom areas to the corresponding elements of the dataset and perform one step of parameters learning as described in Eqs. 4 and 5.

We then test the capacity of our network to classify data by only clamping the bottom level to the data and letting the top-level activity follow Eq. 2. We select as the output class index the index of the maximum top-level activity and plot the corresponding classification in Fig. 4dii. For comparison, we also plot in Fig. 4diii the classification results obtained with the same 2x2 architecture but using classical predictive coding dynamics and following the same training and testing procedures. In Fig. 4e, we plot the associated performance, with the addition of the maximum-likelihood estimate with perfect knowledge of the means and variances.

Simulations and pseudocodes for confidence learning and Bayes-optimal integration in dynamic environments are reported in Supplementary Material, SI5 and SI6.

## Supplementary Material

pgae404_Supplementary_Data

## Data Availability

There are no data underlying this work.

## Preprints

This manuscript was posted on a preprint: https://doi.org/10.48550/arXiv.2309.16046.
