# Correction to: Confidence and second-order errors in cortical circuits

**DOI:** 10.1093/pnasnexus/pgaf217

**Published:** 2025-07-21

This is a correction to: Arno Granier, Mihai A Petrovici, Walter Senn, Katharina A Wilmes, Confidence and second-order errors in cortical circuits, *PNAS Nexus*, Volume 3, Issue 9, September 2024, pgae404, https://doi.org/10.1093/pnasnexus/pgae404

The funding section has been updated to acknowledge support from the European Union’s Horizon Europe Programme under the Specific Grant Agreement No. 101147319 (EBRAINS 2.0 Project).

