# Supplementary material for: Confidence and second-order errors in cortical circuits
Source: PNAS Nexus. 2024 Sep 13;3(9):pgae404. doi: 10.1093/pnasnexus/pgae404 (PMC11437657; doi:10.1093/pnasnexus/pgae404)
Supplement: pgae404_Supplementary_Data [file pgae404_supplementary_data.pdf]

PNAS nexus

Supplementary Information for

Confidence and second-order errors in cortical circuits

Arno Granier, Mihai A. Petrovici, Walter Senn, and Katharina A. Wilmes

Corresponding Author: Arno Granier.
E-mail: arno.granier@unibe.ch

This PDF file includes:

Supplementary text
Figs. S1 to S4
SI References

Supplementary Information Text

1. Energy

The density of a multivariate Gaussian with diagonal covariance $\boldsymbol{\Sigma} = \mathrm{diag}(\boldsymbol{\sigma}^2)$ with $\boldsymbol{\sigma}^2 > 0$ is

$$f(\boldsymbol{u};\boldsymbol{\mu},\boldsymbol{\sigma}^2) = (2\pi)^{-k/2}\det(\boldsymbol{\Sigma})^{-1/2}\exp\left(-\frac{1}{2}(\boldsymbol{u}-\boldsymbol{\mu})^T\boldsymbol{\Sigma}^{-1}(\boldsymbol{u}-\boldsymbol{\mu})\right) \quad \text{[S1]}$$

$$= (2\pi)^{-k/2}\left(\prod_i \boldsymbol{\pi}_i\right)^{1/2}\exp\left(-\frac{1}{2}\|\boldsymbol{e}\|^2_{\boldsymbol{\pi}}\right)\ , \quad \text{[S2]}$$

noting $\boldsymbol{\pi} = \mathbf{1}/\boldsymbol{\sigma}^2$ where the division is taken elementwise and $\|\boldsymbol{e}\|^2_{\boldsymbol{\pi}} = \|\boldsymbol{u}-\boldsymbol{\mu}\|^2_{\boldsymbol{\Sigma}^{-1}} = (\boldsymbol{u}-\boldsymbol{\mu})^T\boldsymbol{\Sigma}^{-1}(\boldsymbol{u}-\boldsymbol{\mu})$. For the determinant, remark that the determinant of diagonal matrix is the product of its diagonal elements.
We now derive Eq. 8

$$-\log p(\boldsymbol{u_0},\boldsymbol{u_1},\ldots,\boldsymbol{u_n}) = -\log\left(K\prod_{l=0}^{n-1}p(\boldsymbol{u_\ell}|\boldsymbol{u_{\ell+1}})\right) \quad \text{[S3]}$$

$$= -\sum_{l=0}^{n-1}\log p(\boldsymbol{u_\ell}|\boldsymbol{u_{\ell+1}}) + K \quad \text{[S4]}$$

$$= -\sum_{l=0}^{n-1}\log\left((2\pi)^{-k_\ell/2}\left(\prod_i(\boldsymbol{\pi_\ell})_i\right)^{1/2}\exp\left(-\frac{1}{2}\|\boldsymbol{e_\ell}\|^2_{\boldsymbol{\pi_\ell}}\right)\right) + K \quad \text{[S5]}$$

$$= -\sum_{l=0}^{n-1}\log\left((2\pi)^{-k_\ell/2}\right) - \frac{1}{2}\sum_{l=0}^{n-1}\log\left(\prod_i(\boldsymbol{\pi_\ell})_i\right) + \frac{1}{2}\sum_{l=0}^{n-1}\|\boldsymbol{e_\ell}\|^2_{\boldsymbol{\pi_\ell}} + K \quad \text{[S6]}$$

$$= \frac{1}{2}\sum_{l=0}^{n-1}\|\boldsymbol{e_\ell}\|^2_{\boldsymbol{\pi_\ell}} - \frac{1}{2}\sum_{l=0}^{n-1}\log|\boldsymbol{\pi_\ell}| + K\ , \quad \text{[S7]}$$

where to get Eq. S3 we used Eq. 6 and to get Eq. S5 we used Eqs. 7 and S2.

2. Partial derivatives of the energy

We now give a high-level view of the derivation of partial derivatives of the energy E used in neuronal and synaptic dynamics Eqs. 2, 4 and 5. We omit calculation details for the sake of brevity. As a reminder, we set $\boldsymbol{e_\ell} = \boldsymbol{u_\ell} - \boldsymbol{W_\ell}\phi(\boldsymbol{u_{\ell+1}})$, $\boldsymbol{\pi_\ell} = \boldsymbol{A_\ell}\phi(\boldsymbol{u_{\ell+1}})$, $\boldsymbol{\delta_\ell} = (\boldsymbol{\pi_\ell}^{-1} - \boldsymbol{e}_\ell^2)/2$ and $\circ$ is the componentwise (Hadamard) product.
For this, we will make use of the following matrix calculus formulas:

$$\forall \boldsymbol{M}\text{ symmetric}\ , \frac{\partial \boldsymbol{x}^T\boldsymbol{M}\boldsymbol{x}}{\partial \boldsymbol{x}} = 2\boldsymbol{M}\boldsymbol{x}\ , \quad \text{[i]}$$

$$\frac{\partial \boldsymbol{g}(\boldsymbol{x})}{\partial \boldsymbol{x}} = \frac{\partial g(f(\boldsymbol{x}))}{\partial f(\boldsymbol{x})}\frac{\partial f(\boldsymbol{x})}{\partial \boldsymbol{x}}\ \ \text{(chain rule)}\ , \quad \text{[ii]}$$

$$\frac{\partial \mathbf{1}^T\log(\boldsymbol{M}\boldsymbol{x})}{\partial \boldsymbol{x}} = \mathbf{1}^T(\mathrm{diag}(\mathbf{1}/(\boldsymbol{M}\boldsymbol{x}))\boldsymbol{M}) = \boldsymbol{M}^T(\mathbf{1}/(\boldsymbol{M}\boldsymbol{x}))\ \text{(with the division being componentwise)}\ , \quad \text{[iii]}$$

$$\frac{\partial f(\boldsymbol{x})^T g(\boldsymbol{x})}{\partial \boldsymbol{x}} = \frac{\partial f(\boldsymbol{x})}{\partial \boldsymbol{x}}g(\boldsymbol{x}) + \frac{\partial g(\boldsymbol{x})}{\partial \boldsymbol{x}}f(\boldsymbol{x})\ , \quad \text{[iv]}$$

$$\frac{\partial f(\boldsymbol{x})\circ g(\boldsymbol{x})}{\partial \boldsymbol{x}} = \frac{\partial f(\boldsymbol{x})}{\partial \boldsymbol{x}}\mathrm{diag}(g(\boldsymbol{x})) + \frac{\partial g(\boldsymbol{x})}{\partial \boldsymbol{x}}\mathrm{diag}(f(\boldsymbol{x}))\ . \quad \text{[v]}$$

Latent variables. The derivative with respect to $\boldsymbol{u_\ell}$ can be decomposed in three terms

$$2\frac{\partial E}{\partial \boldsymbol{u_\ell}} = \frac{\partial\|\boldsymbol{e_\ell}\|^2_{\boldsymbol{\pi_\ell}}}{\partial \boldsymbol{u_\ell}} + \frac{\partial\|\boldsymbol{e_{\ell-1}}\|^2_{\boldsymbol{\pi_{\ell-1}}}}{\partial \boldsymbol{u_\ell}} - \frac{\partial\log|\boldsymbol{\pi_{\ell-1}}|}{\partial \boldsymbol{u_\ell}}\ . \quad \text{[S8]}$$

We compute those three terms independently.
For the first term, the derivation is straightforward and follow directly from (i) and (ii)

$$\frac{\partial\|\boldsymbol{e_\ell}\|^2_{\boldsymbol{\pi_\ell}}}{\partial \boldsymbol{u_\ell}} = 2\boldsymbol{\pi_\ell}\circ\boldsymbol{e_\ell}\ . \quad \text{[S9]}$$

For the second term we first remark that it can be written as $\frac{\partial \boldsymbol{e}_{\boldsymbol{\ell}-\mathbf{1}}^T(\boldsymbol{\pi}_{\boldsymbol{\ell}-\mathbf{1}} \circ \boldsymbol{e}_{\boldsymbol{\ell}-\mathbf{1}})}{\partial \boldsymbol{u}_{\boldsymbol{\ell}}}$, apply (iv) and then develop $\frac{\partial \boldsymbol{\pi}_{\boldsymbol{\ell}-\mathbf{1}} \circ \boldsymbol{e}_{\boldsymbol{\ell}-\mathbf{1}}}{\partial \boldsymbol{u}_{\boldsymbol{\ell}}}$ following (v)

$$\frac{\partial \|\boldsymbol{e}_{\boldsymbol{\ell}-\mathbf{1}}\|^2_{\boldsymbol{\pi}_{\boldsymbol{\ell}-\mathbf{1}}}}{\partial \boldsymbol{u}_{\boldsymbol{\ell}}} = -2\phi'(\boldsymbol{u}_{\boldsymbol{\ell}}) \circ \left(\boldsymbol{W}^T_{\boldsymbol{\ell}-\mathbf{1}} (\boldsymbol{\pi}_{\boldsymbol{\ell}-\mathbf{1}} \circ \boldsymbol{e}_{\boldsymbol{\ell}-\mathbf{1}}) - \frac{1}{2} \boldsymbol{A}^T_{\boldsymbol{\ell}-\mathbf{1}} \boldsymbol{e}^2_{\boldsymbol{\ell}-\mathbf{1}} \right) . \tag{S10}$$

For the third term remark that $\log |\boldsymbol{x}| = \mathbf{1}^T \log \boldsymbol{x}$, then a straightforward application of (iii) is sufficient

$$\frac{\partial \log |\boldsymbol{\pi}_{\boldsymbol{\ell}-\mathbf{1}}|}{\partial \boldsymbol{u}_{\boldsymbol{\ell}}} = \phi'(\boldsymbol{u}_{\boldsymbol{\ell}}) \circ \boldsymbol{A}^T_{\boldsymbol{\ell}-\mathbf{1}} \boldsymbol{\pi}^{-1}_{\boldsymbol{\ell}-\mathbf{1}} . \tag{S11}$$

Finally putting it all together we have

$$\frac{\partial E}{\partial \boldsymbol{u}_{\boldsymbol{\ell}}} = \boldsymbol{\pi}_{\boldsymbol{\ell}} \circ \boldsymbol{e}_{\boldsymbol{\ell}} - \phi'(\boldsymbol{u}_{\boldsymbol{\ell}}) \circ \left(\boldsymbol{W}^T_{\boldsymbol{\ell}-\mathbf{1}} (\boldsymbol{\pi}_{\boldsymbol{\ell}-\mathbf{1}} \circ \boldsymbol{e}_{\boldsymbol{\ell}-\mathbf{1}}) + \boldsymbol{A}^T_{\boldsymbol{\ell}-\mathbf{1}} \boldsymbol{\delta}_{\boldsymbol{\ell}-\mathbf{1}} \right) . \tag{S12}$$

Prediction weights. The derivative with respect to $\boldsymbol{W}_{\boldsymbol{\ell}}$ is simply

$$2\frac{\partial E}{\partial \boldsymbol{W}_{\boldsymbol{\ell}}} = \frac{\partial \|\boldsymbol{e}_{\boldsymbol{\ell}}\|^2_{\boldsymbol{\pi}_{\boldsymbol{\ell}}}}{\partial \boldsymbol{W}_{\boldsymbol{\ell}}} . \tag{S13}$$

The derivation is straightforward and follow directly from (i) and (ii)

$$\frac{\partial \|\boldsymbol{e}_{\boldsymbol{\ell}}\|^2_{\boldsymbol{\pi}_{\boldsymbol{\ell}}}}{\partial \boldsymbol{W}_{\boldsymbol{\ell}}} = \frac{\partial \boldsymbol{e}^T_{\boldsymbol{\ell}} \mathrm{diag}(\boldsymbol{\pi}_{\boldsymbol{\ell}}) \boldsymbol{e}_{\boldsymbol{\ell}}}{\partial \boldsymbol{e}_{\boldsymbol{\ell}}} \frac{\partial \boldsymbol{e}_{\boldsymbol{\ell}}}{\partial \boldsymbol{W}_{\boldsymbol{\ell}}} = -2(\boldsymbol{\pi}_{\boldsymbol{\ell}} \circ \boldsymbol{e}_{\boldsymbol{\ell}}) \phi(\boldsymbol{u}_{\boldsymbol{\ell}+\mathbf{1}})^T \tag{S14}$$

and

$$\frac{\partial E}{\partial \boldsymbol{W}_{\boldsymbol{\ell}}} = -(\boldsymbol{\pi}_{\boldsymbol{\ell}} \circ \boldsymbol{e}_{\boldsymbol{\ell}}) \phi(\boldsymbol{u}_{\boldsymbol{\ell}+\mathbf{1}})^T . \tag{S15}$$

Confidence estimation weights. The derivative with respect to $\boldsymbol{W}_{\boldsymbol{\ell}}$ can be decomposed in two terms

$$2\frac{\partial E}{\partial \boldsymbol{A}_{\boldsymbol{\ell}}} = \frac{\partial \|\boldsymbol{e}_{\boldsymbol{\ell}}\|^2_{\boldsymbol{\pi}_{\boldsymbol{\ell}}}}{\partial \boldsymbol{A}_{\boldsymbol{\ell}}} - \frac{\partial \log |\boldsymbol{\pi}_{\boldsymbol{l}}|}{\partial \boldsymbol{A}_{\boldsymbol{\ell}}} . \tag{S16}$$

We compute those two terms independently. For these we find it easier to compute derivatives element by element. For the first term remark that $\|\boldsymbol{e}_{\boldsymbol{\ell}}\|^2_{\boldsymbol{\pi}_{\boldsymbol{\ell}}} = \sum_i \left(\boldsymbol{e}_{\boldsymbol{\ell}}{}^2\right)_i \sum_j (\boldsymbol{A}_{\boldsymbol{\ell}})_{i,j} (\phi(\boldsymbol{u}_{\boldsymbol{\ell}+\mathbf{1}}))_j$ and then it is simple to see that

$$\frac{\partial \|\boldsymbol{e}_{\boldsymbol{\ell}}\|^2_{\boldsymbol{\pi}_{\boldsymbol{\ell}}}}{\partial (\boldsymbol{A}_{\boldsymbol{\ell}})_{i,j}} = \left(\boldsymbol{e}_{\boldsymbol{\ell}}{}^2\right)_i (\phi(\boldsymbol{u}_{\boldsymbol{\ell}+\mathbf{1}}))_j . \tag{S17}$$

For the second term remark that $\log |\boldsymbol{\pi}_{\boldsymbol{l}}| = \sum_i \log(\boldsymbol{\pi}_{\boldsymbol{\ell}})_i = \sum_i \log \left(\sum_j (\boldsymbol{A}_{\boldsymbol{\ell}})_{i,j} (\phi(\boldsymbol{u}_{\boldsymbol{\ell}+\mathbf{1}}))_j \right)$, and then

$$\frac{\partial \log |\boldsymbol{\pi}_{\boldsymbol{l}}|}{\partial (\boldsymbol{A}_{\boldsymbol{\ell}})_{i,j}} = \frac{(\phi(\boldsymbol{u}_{\boldsymbol{\ell}+\mathbf{1}}))_j}{\sum_j (\boldsymbol{A}_{\boldsymbol{\ell}})_{i,j} (\phi(\boldsymbol{u}_{\boldsymbol{\ell}+\mathbf{1}}))_j} = \left(\boldsymbol{\pi}^{-1}_{\boldsymbol{\ell}}\right)_i (\phi(\boldsymbol{u}_{\boldsymbol{\ell}+\mathbf{1}}))_j . \tag{S18}$$

Putting it together and writing it in matrix form

$$\frac{\partial E}{\partial \boldsymbol{A}_{\boldsymbol{\ell}}} = -\boldsymbol{\delta}_{\boldsymbol{\ell}} \phi(\boldsymbol{u}_{\boldsymbol{\ell}+\mathbf{1}})^T . \tag{S19}$$

Latent variables - second derivative. The second derivative of the energy with respect to latent representations is

$$\begin{aligned} \frac{\partial^2 E}{\partial \boldsymbol{u}^2_{\boldsymbol{\ell}}} =& \mathrm{diag}(\boldsymbol{\pi}_{\boldsymbol{\ell}}) - \mathrm{diag}(\boldsymbol{r}''_{\boldsymbol{\ell}} \circ (\boldsymbol{W}^T_{\boldsymbol{\ell}-\mathbf{1}} (\boldsymbol{\pi}_{\boldsymbol{\ell}-\mathbf{1}} \circ \boldsymbol{e}_{\boldsymbol{\ell}-\mathbf{1}}) + \boldsymbol{A}^T_{\boldsymbol{\ell}-\mathbf{1}} \boldsymbol{\delta}_{\boldsymbol{\ell}-\mathbf{1}})) \\ &+ \mathrm{diag}(\boldsymbol{r}'_{\boldsymbol{\ell}})(\boldsymbol{W}^T_{\boldsymbol{\ell}-\mathbf{1}} \mathrm{diag}(\boldsymbol{e}_{\boldsymbol{\ell}-\mathbf{1}}) \boldsymbol{A}_{\boldsymbol{\ell}-\mathbf{1}} - \boldsymbol{W}^T_{\boldsymbol{\ell}-\mathbf{1}} \mathrm{diag}(\boldsymbol{\pi}_{\boldsymbol{\ell}-\mathbf{1}}) \boldsymbol{W}_{\boldsymbol{\ell}-\mathbf{1}} \\ &- 0.5 \boldsymbol{A}^T_{\boldsymbol{\ell}-\mathbf{1}} \mathrm{diag}(\boldsymbol{\pi}^{-2}_{\boldsymbol{\ell}-\mathbf{1}}) \boldsymbol{A}_{\boldsymbol{\ell}-\mathbf{1}} + \boldsymbol{A}^T_{\boldsymbol{\ell}-\mathbf{1}} \mathrm{diag}(\boldsymbol{e}_{\boldsymbol{\ell}-\mathbf{1}}) \boldsymbol{W}^T_{\boldsymbol{\ell}-\mathbf{1}}) \end{aligned} \tag{S20}$$

3. Metrics for parameter learning

In this work, we took confidence as a metric when deriving neuronal dynamics (Eq. 2) but used the default Euclidean metric when deriving synaptic learning rules (Eqs. 4 and 5). The same approach of taking as a metric an approximate second-order derivative in gradient descent could be used not only for inference but also (and in fact more classically) for parameter learning. In that case, second derivatives are also expressed with confidence/variance:

$$-\frac{\partial^2 \log f(\boldsymbol{u}; \boldsymbol{m}, \boldsymbol{p}^{-1})}{\partial \boldsymbol{m}^2} = \mathrm{diag}(\boldsymbol{p}) , \tag{S21}$$

$$-\frac{\partial^2 \log f(\boldsymbol{u}; \boldsymbol{m}, \boldsymbol{p}^{-1})}{\partial \boldsymbol{p}^2} = \mathrm{diag}(\boldsymbol{p}^{-2}) . \tag{S22}$$

with f being the density of a multivariate Gaussian with mean $\boldsymbol{m}$ and variance $\boldsymbol{p}^{-1}$ and $\boldsymbol{m}, \boldsymbol{p}$ not functions of $\boldsymbol{u}$. Note that this is not always expressed correctly in the literature (e.g., (1, 2) Eq. 64, where the Fisher information matrix $\mathcal{G}$ instead of its inverse appears in the definition of natural gradient, c.f. our Eq. 8), leading to confusion on the link between confidence weighting and natural gradient descent.

4. Intuition at equilibrium in the linear case

At equilibrium of Eq. 2, noting $\boldsymbol{\Pi_k} = \text{diag}(\boldsymbol{\pi_k})$, ignoring second-order errors ($\boldsymbol{\delta_{\ell-1}} = 0$) and working in the linear case $\phi(\boldsymbol{x}) = \boldsymbol{x}$ we have the value at equilibrium

$$\boldsymbol{u}^*_{\boldsymbol{\ell}} = (\boldsymbol{\Pi_\ell} + \boldsymbol{W}^T_{\boldsymbol{\ell-1}}\boldsymbol{\Pi_{\ell-1}}\boldsymbol{W_{\ell-1}})^{-1}(\boldsymbol{\Pi_\ell}\boldsymbol{W_\ell}\boldsymbol{u_{\ell+1}} + \boldsymbol{W}^T_{\boldsymbol{\ell-1}}\boldsymbol{\Pi_{\ell-1}}\boldsymbol{u_{\ell-1}}) \ , \tag{S23}$$

where the first term can be interpreted as a normalization factor and the second term as a weighted sum of higher and lower representations "translated in the language" of the local level l through prediction weight matrices. Remark that, if the confidence $\boldsymbol{\Pi_{\ell-1}}$ of the prediction that level l makes about level $l-1$ is negligible compared to the confidence $\boldsymbol{\Pi_l}$ of the prediction that level $l+1$ makes about level l, which we will note $\boldsymbol{\Pi_{\ell-1}}/\boldsymbol{\Pi_l} \to 0$, then the activity of level l goes to the prediction made by level $l+1$ (the prior)

$$\boldsymbol{\Pi_{\ell-1}}/\boldsymbol{\Pi_l} \to 0 \implies \boldsymbol{u}^*_{\boldsymbol{\ell}} \to \boldsymbol{W_\ell}\boldsymbol{u_{\ell+1}} \ . \tag{S24}$$

Inversely, when the prediction that level $l+1$ makes about what the activity in level l should be (the prior) is deemed unreliable compared to the prediction that level l makes about what the activity of level $l-1$ should be, then the activity of level l goes to a value such that its prediction is the activity in level $l-1$

$$\boldsymbol{\Pi_l}/\boldsymbol{\Pi_{\ell-1}} \to 0 \implies \boldsymbol{W_{\ell-1}}\boldsymbol{u}^*_{\boldsymbol{\ell}} \to \boldsymbol{u_{\ell-1}} \ . \tag{S25}$$

5. Simulation details: Confidence learning

For simulations presented in Fig. S3c, we follow the simulation setup presented in Fig. S3a and described in more details below and in Supplementary Algorithm 1.

We consider a higher area with $N_{\ell+1}$ neurons and a lower area with N_ℓ neurons. We consider N_c different classes of inputs, each with its own distribution $\mathcal{N}(\boldsymbol{\mu_i}, \boldsymbol{\sigma_i^2}), i \in [1, N_c]$, where $\boldsymbol{\mu_i}$ and $\boldsymbol{\sigma_i^2}$ are vectors of size N_ℓ. We initialize all $\boldsymbol{\mu_i}$ following a $\mathcal{U}(-1, 1)$ and all $\boldsymbol{\sigma}_i^2$ following a $\mathcal{U}(1/4, 1)$. Then we choose the representational mode of the higher area, either random binary vectors or one-hot encoded and initialize higher-level representations $\boldsymbol{r_i}, i \in [1, N_c]$ as random binary vectors of size $N_{\ell+1}$ with on average p ones or one-hot encoded i in $N_{\ell+1}$, respectively. The confidence estimation matrix $\boldsymbol{A}$ is then initialized as a matrix filled with α, with $\alpha = 1/pN_{\ell+1}$ for the random binary vector case and $\alpha = 1$ for the one-hot encoded case. We then repeat the following procedure for multiple epochs:

1. For each class, sample a data point $\boldsymbol{x_i}$ from $\mathcal{N}(\boldsymbol{\mu_i}, \boldsymbol{\sigma_i^2})$.
2. Set the higher-level representation to $\boldsymbol{r_i}$.
3. Compute the confidence estimate $\boldsymbol{\pi_i} = \boldsymbol{A}\boldsymbol{r_i}$.
4. Compute the second-order error $\boldsymbol{\delta_i} = (1/\boldsymbol{\pi_i} - (\boldsymbol{x_i} - \boldsymbol{\mu_i})^2)/2$.
5. Update $\boldsymbol{A}$ following Eq. 9.

In Fig. S3c, we plot the evolution of $(\sqrt{N_\ell}N_c)^{-1}\sum_i \|\boldsymbol{\sigma_i^2} - 1/\boldsymbol{A}\boldsymbol{r_i}\|$ through epochs. For Fig. S3c, parameters are $T = 10000, N_{\ell+1} = N_\ell = 100, \eta = 0.001$ with N_c varying depending on the simulation. A similar procedure is used for Fig. S3b, but following Eq. 4.

Supporting Algorithm 1 Confidence learning

Require: T, $N_{\ell+1}$ N_ℓ, N_c, η, overlap, p
  $\boldsymbol{\sigma}^2 = [1.5 \text{ rand}(N_\ell) + 0.5$ for _ in 1:$N_c]$ ▷ $\boldsymbol{\sigma}^2$ initialization, random uniform between 1/2 and 2
  $\boldsymbol{\mu} = [2 \text{ rand}(N_\ell) - 1$ for _ in 1:$N_c]$ ▷ $\boldsymbol{\mu}$ initialization, random uniform between -1 and 1
  if overlap **then**
    $\boldsymbol{r} = [\text{rand}(N_{\ell+1}) < p$ for _ in 1:$N_c]$ ▷ $\boldsymbol{r}$ initialization, random binary vector with p% ones on average
    $\boldsymbol{A} = \text{ones}(N_\ell, N_{\ell+1})/(pN_{\ell+1})$ ▷ $\boldsymbol{A}$ initialization (such that the mean starting $\boldsymbol{\pi}$ is one)
  else
    $\boldsymbol{r}$ = [[(j==i) ? 1 : 0 for j in 1:$N_{\ell+1}$] for i in 1:N_c] ▷ $\boldsymbol{r}$ initialization, onehot encoded
    $\boldsymbol{A} = \text{ones}(N_\ell, N_{\ell+1})$ ▷ $\boldsymbol{A}$ initialization (such that the mean starting $\boldsymbol{\pi}$ is one)
  store = []
  for t in 1:T **do**
    for i in 1:N_c **do**
      $\boldsymbol{x} \sim \mathcal{N}(\boldsymbol{\mu}[\text{i}], \boldsymbol{\sigma}^2[\text{i}])$ ▷ sample lower level data
      $\boldsymbol{\pi} = \boldsymbol{A}\boldsymbol{r}[\text{i}]$ ▷ Compute confidence estimate
      $\boldsymbol{\delta} = 0.5(\boldsymbol{1}/\boldsymbol{\pi} - (\boldsymbol{x} - \boldsymbol{\mu}[\text{i}])^2)$ ▷ compute second-order errors
      $\boldsymbol{A} \leftarrow \boldsymbol{A} + \eta\boldsymbol{A} \circ \boldsymbol{\delta}\boldsymbol{r}[i]^T$ ▷ update $\boldsymbol{A}$ following Eq. 5
    store[t] = sum(norm([$\boldsymbol{\sigma}^2$[i] - $\boldsymbol{1}/\boldsymbol{A}\boldsymbol{r}$[i] for i in 1:$N_c$]))/($\sqrt{N_\ell}N_c$) ▷ distance between (real) $\boldsymbol{\sigma}^2$ and $\boldsymbol{1}/\boldsymbol{\pi}$

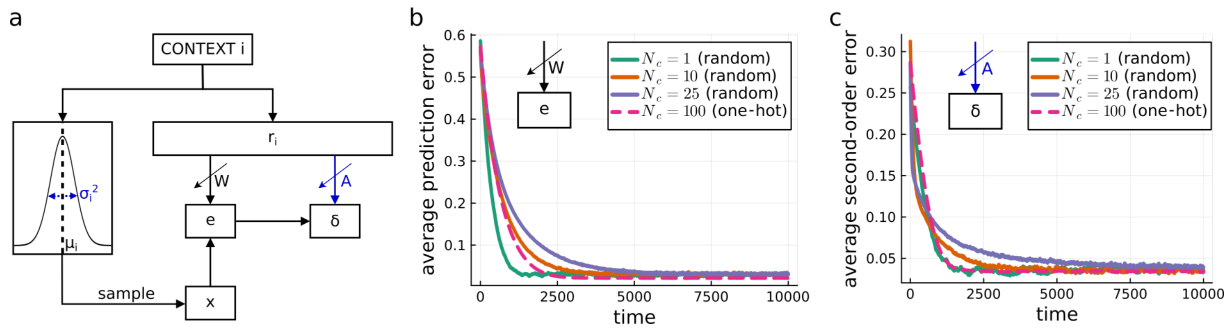

Fig. S1. Error-correcting synaptic learning. (a) In these simulations, we consider a higher area with $N_{\ell+1}$ neurons and a lower area with N_ℓ neurons. Specifically, here we take $N_{\ell+1} = N_\ell = 100$. The activity vector in the higher area can take N_c different values [$\boldsymbol{r_n},\ n=1,\ldots,N_c$], to each of which is associated a different mean [$\boldsymbol{\mu_n}$] and a different variance [$\boldsymbol{\sigma_n^2}$]. The activity in the lower area is then sampled from the Gaussian distribution with this mean and variance. Predictions [$\boldsymbol{Wr_i}$] and confidence estimates [$\boldsymbol{Ar_i}$] are formed from the higher-level representation and prediction errors [$\boldsymbol{e} = \boldsymbol{x} - \boldsymbol{Wr_i}$] and second-order errors [$\boldsymbol{\delta} = \boldsymbol{1}/\boldsymbol{Ar_i} - \boldsymbol{e}^2$] are computed and used to learn parameters [$\boldsymbol{W}$ and $\boldsymbol{A}$]. For simulations marked `(random)`, higher-level representations are random binary vectors with an average of 50% of ones. For simulations marked `(one-hot)`, higher-level representations are one-hot encoded. (b) Here we show that with the learning rule Eq. 4 the network correctly learns to estimate the means [$\boldsymbol{\mu_n},\ n=1,\ldots,N_c$] from higher-level activity [$\boldsymbol{r_n},\ n=1,\ldots,N_c$]. In these simulations we suppose that the confidence estimate is 1. (c) Here we show that with the learning rule Eq. 5 the network correctly learns to estimate the confidences [$\boldsymbol{1}/\boldsymbol{\sigma_n^2}$] from higher-level activity [$\boldsymbol{r_n}$].

6. Simulation details: Approximate Bayes-optimal integration

For simulations presented in Fig. S2b, we follow the simulation procedure described below. Pseudocode for these simulations is presented in Supplementary Algorithm 2 and a mathematical intuition is given in S4a.

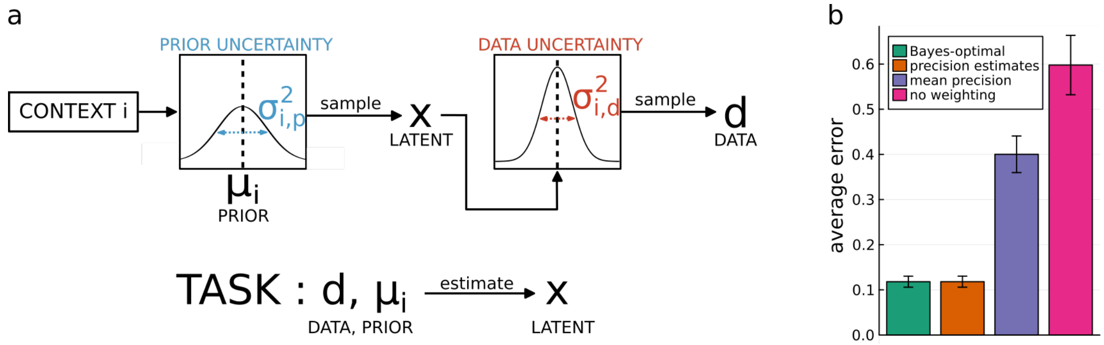

Fig. S2. Approximate Bayes-optimal computation in a volatile environment. (a) Simulation setup. (b) Simulation results for data and prior integration when the distributiosn of both data and prior are context-dependent. Bayes-optimal (green): a Bayes-optimal estimate, with knowledge of true prior uncertainty and true data confidence. Precision estimates (orange): our dynamics, with knowledge of true prior uncertainty and an estimate of data confidence as a function of current representation. Mean confidence (purple): an estimate with knowledge only of the mean prior uncertainty and data confidence across contexts. No weighting (magenta): an estimate blind to uncertainty and confidence. We plot the average distance between each estimate and the true latent. The error bars indicate the standard deviation.

We consider a higher area with $N_{\ell+1}$ neurons and a lower area with N_ℓ neurons. We consider N_c different classes of inputs, each with its own distribution $\mathcal{N}(\boldsymbol{\mu_i}, \boldsymbol{\sigma_i^2}), i \in [1, N_c]$, where $\boldsymbol{\mu_i}$ and $\boldsymbol{\sigma_i^2}$ are vectors of size N_ℓ. We initialize all $\boldsymbol{\mu_i}$ following a $\mathcal{U}(0, 2/N_\ell)$ and all $\boldsymbol{\sigma_i^2}$ by randomly choosing each component in $\{0.1, 2\}$ with a 50% chance. We initialize the confidence estimation matrix $\boldsymbol{A}$ following a $\mathcal{U}(0,2)$. We additionally collect the mean prior variance vector across classes $\bar{\boldsymbol{\sigma}}^2 = \frac{1}{N_c}\sum_i \boldsymbol{\sigma_i^2}$ and the mean data confidence vector across classes $\bar{\boldsymbol{\pi}} = \frac{1}{N_c}\sum_i \boldsymbol{A}\phi(\boldsymbol{\mu_i})$. We then repeat across epochs the following procedure. For each class i (1) we sample a true target latent $\boldsymbol{x} \sim \mathcal{N}(\boldsymbol{\mu_i}, \boldsymbol{\sigma_i^2})$. We consider that the confidence estimation weights are correct such that the confidence of the data is $\boldsymbol{\pi} = \boldsymbol{A}\phi(\boldsymbol{x})$. (2) Then we sample noisy data. Here we want to focus on confidence estimation and not mean prediction, so we suppose that the prediction function is the identity, and we then sample data $\boldsymbol{d} \sim \mathcal{N}(\boldsymbol{x}, 1/\boldsymbol{\pi})$. The goal is then to infer $\boldsymbol{x}$ from data $\boldsymbol{d}$ and prior $\boldsymbol{\mu_i}$. We do that in four different ways that differ in how they take into account uncertainty and confidence:

(3i) a Bayes-optimal estimate, with knowledge of true prior variance and true data confidence

$$\boldsymbol{u} = (\boldsymbol{\pi} \circ \boldsymbol{d} + \boldsymbol{\sigma_i^2} \circ \boldsymbol{\mu_i})/(\boldsymbol{\pi} + \boldsymbol{\sigma_i}^{-2}) \ . \tag{S26}$$

(3ii) our dynamics, with knowledge of true prior variance and data confidence estimation

$$\tau \dot{\boldsymbol{u}} = -\boldsymbol{u} + \boldsymbol{\mu_i} + \boldsymbol{\sigma_i^2} \circ \boldsymbol{A}\phi(\boldsymbol{u}) \circ (\boldsymbol{d} - \boldsymbol{u}) \ . \tag{S27}$$

(3iii) an estimate with knowledge only of the mean prior variance and data confidence across classes

$$\tau \dot{\boldsymbol{u}} = -\boldsymbol{u} + \boldsymbol{\mu_i} + \bar{\boldsymbol{\sigma}} \circ \bar{\boldsymbol{\pi}} \circ (\boldsymbol{d} - \boldsymbol{u}) \ . \tag{S28}$$

(3iv) an estimate blind to variance and confidence

$$\tau \dot{\boldsymbol{u}} = -\boldsymbol{u} + \boldsymbol{\mu_i} + (\boldsymbol{d} - \boldsymbol{u}) \ . \tag{S29}$$

In Fig. S2b, we plot the average distance between each estimate and the true latent $(N_c N_e \sqrt{N_\ell})^{-1} \|x - u\|$ and its standard deviation.

Supporting Algorithm 2 Approximate Bayes-optimal integration

Require: $N_{\ell+1}$, N_ℓ, N_c ϕ, τ, T, N_e
  $\boldsymbol{\sigma}^2 = [\text{choice}([0.1, 2])$ for _ in $1 : N_l]$ for _ in $1{:}N_c]$ ▷ Initialize prior variance
  $\boldsymbol{\mu} = [2\text{rand}(N_\ell)/N_\ell$ for _ in $1{:}N_c]$ ▷ Initialize prior mean
  $\boldsymbol{A} = 2\text{rand}(N_\ell, N_{\ell+1})$ ▷ Initialize confidence estimation weights
  $\bar{\boldsymbol{\sigma}} = \text{sum}(\boldsymbol{\sigma^2})/N_c$ ▷ mean prior variance
  $\bar{\boldsymbol{\pi}} = \text{sum}([\boldsymbol{A}\boldsymbol{\mu}[i]$ for i in $1 : N_c])/N_c$ ▷ mean data confidence
  err1s, err2s, err3s, err4s = [ ], [ ], [ ], [ ]
  for t in $1{:}N_e$ **do**
    for i in 1 N_c **do**
      $\boldsymbol{x} \sim \mathcal{N}(\boldsymbol{\mu}[i], \boldsymbol{\sigma}^2[i])$ ▷ sample true data
      $\boldsymbol{\pi} = \boldsymbol{A}\phi(\boldsymbol{x})$ ▷ compute confidence estimate at true data
      $\boldsymbol{d} \sim \mathcal{N}(\boldsymbol{x}, 1/\boldsymbol{\pi})$ ▷ sample noisy data
      $\hat{\boldsymbol{x}} = (\boldsymbol{\pi} \circ \boldsymbol{d} + \boldsymbol{\sigma}^{-2}[i] \circ \boldsymbol{\mu}[i])/(\boldsymbol{\pi} + \boldsymbol{\sigma}^{-2}[i])$ ▷ Bayes-optimal estimate
      err1s.append(norm$(\boldsymbol{x} - \hat{\boldsymbol{x}})/\sqrt{N_\ell}$)
      $\boldsymbol{u} = \boldsymbol{1}$
      for t in 1:T **do**
        $\boldsymbol{u}$ += $(1/\tau) * (-\boldsymbol{u} + \boldsymbol{\mu}[i] + \boldsymbol{\sigma}^2[i] \circ \boldsymbol{A}\phi(\boldsymbol{u}) \circ (\boldsymbol{d} - \boldsymbol{u}))$ ▷ dynamics with confidence estimation
      err2s.append(norm$(\boldsymbol{x} - \boldsymbol{u})/\sqrt{N_\ell}$)
      $\boldsymbol{u} = \boldsymbol{1}$
      for t in 1:T **do**
        $\boldsymbol{u}$ += $(1/\tau) * (-\boldsymbol{u} + \boldsymbol{\mu}[i] + \bar{\boldsymbol{\sigma}} \circ \bar{\boldsymbol{\pi}} \circ (\boldsymbol{d} - \boldsymbol{u}))$ ▷ dynamics with average confidence and prior variance
      err3s.append(norm$(\boldsymbol{x} - \boldsymbol{u})/\sqrt{N_\ell}$)
      $\boldsymbol{u} = \boldsymbol{1}$
      for t in 1:T **do**
        $\boldsymbol{u}$ += $(1/\tau) * (-\boldsymbol{u} + \boldsymbol{\mu}[i] + (\boldsymbol{d} - \boldsymbol{u}))$ ▷ no weighting
      err4s.append(norm$(\boldsymbol{x} - \boldsymbol{u})/\sqrt{N_\ell}$)

7. Simulation details: Nonlinear binary classification

For simulations presented in Fig. 3 , we built the datasets by sampling $N = 1000$ points $(x_1, y_1), \ldots, (x_N, y_N)$ from each of the Gaussian distributions represented in Fig. 3di (first column: $\mathcal{N}([0,0], \text{diag}([3,3]))$ and $\mathcal{N}([0,0], \text{diag}([1/3, 1/3]))$, second column: $\mathcal{N}([0,0], \text{diag}([1, 1/4]))$ and $\mathcal{N}([0,0], \text{diag}([1/4, 1/4]))$, third column: $\mathcal{N}([1,0], \text{diag}([1/5, 1/5]))$, $\mathcal{N}([-1,0], \text{diag}([1/5, 1/5]))$, $\mathcal{N}([0,1], \text{diag}([1/5, 1/5]))$ and $\mathcal{N}([0,-1], \text{diag}([1/5, 1/5]))$, represented by their 99.7% confidence ellipses) and attaching the corresponding class label (either red or blue).

We then build a 2x2 network where the top level activity is a one-hot representation of the class and the bottom level activity is the coordinate in space (x, y). We train this network in supervised learning settings on the dataset by clamping both top and bottom area to the corresponding elements of the dataset and perform one step of parameters learning as described in Eqs. 4 and 5.

We then test the capacity of our network to classify data by only clamping the bottom level to the data and letting the top level activity follow Eq. 2. We then select as the output class index the index of the maximum top level activity, and plot the corresponding classification in Fig. 4dii.

For comparison, we also plot (in Fig. 4diii) the classification results obtained with the same 2x2 architecture but using classical predictive coding dynamics

$$\tau \dot{\boldsymbol{u}}_{\boldsymbol{\ell}} = -\boldsymbol{u}_{\boldsymbol{\ell}} + \boldsymbol{W}_{\boldsymbol{\ell}} \boldsymbol{r}_{\boldsymbol{\ell+1}} + \boldsymbol{r}'_{\boldsymbol{\ell}} \circ \boldsymbol{W}^{\boldsymbol{T}}_{\boldsymbol{\ell-1}} \boldsymbol{e}_{\boldsymbol{\ell-1}} \ , \tag{S30}$$

$$\dot{\boldsymbol{W}}_{\boldsymbol{\ell}} \propto \boldsymbol{e}_{\boldsymbol{\ell}} \boldsymbol{r}^{T}_{\boldsymbol{\ell+1}} \tag{S31}$$

and following the same training and testing procedures.

In Fig. 4e we plot the associated performance, with the addition of the maximum likelihood estimate with perfect knowledge of the means and variances.

Supporting Algorithm 3 Training

Require: dataset, $\boldsymbol{W}$, $\boldsymbol{A}$, ϕ, η_w, η_a
Ensure: terms of A are strictly positive, range of ϕ is positive
  for $(\boldsymbol{d}, \boldsymbol{t})$ in dataset **do** ▷ $(\boldsymbol{d}, \boldsymbol{t})$ is ([x,y] data, one-hot target)

$\boldsymbol{\pi} = \boldsymbol{A}\phi(\boldsymbol{t})$ ▷ confidence estimate
$\boldsymbol{e} = \boldsymbol{d} - \boldsymbol{W}\phi(\boldsymbol{t})$ ▷ raw error
$\boldsymbol{\delta} = 0.5(\boldsymbol{1}/\boldsymbol{\pi} - \boldsymbol{e}^2)$ ▷ second-order error
$\boldsymbol{W} \leftarrow \boldsymbol{W} + \eta_w(\boldsymbol{\pi} \circ \boldsymbol{e})\boldsymbol{t}^T$ ▷ prediction weight learning, Eq. 4
$\boldsymbol{A} \leftarrow \boldsymbol{A} + \eta_a \boldsymbol{A} \circ \boldsymbol{\delta}\boldsymbol{t}^T$ ▷ confidence estimation weight learning, Eq. 5

Supporting Algorithm 4 Testing

Require: data, $\boldsymbol{W}$, $\boldsymbol{A}$, ϕ, ϕ' τ, T
inferred_labels = dict()
$\boldsymbol{t} = [0.5, 0.5]$ ▷ Uniform initialization of top level
for $\boldsymbol{d}$ in data **do**
for $i = 1..$T **do**
$\boldsymbol{\pi} = \boldsymbol{A}\phi(\boldsymbol{t})$ ▷ confidence estimate
$\boldsymbol{e} = \boldsymbol{d} - \boldsymbol{W}\phi(\boldsymbol{t})$ ▷ raw error
$\boldsymbol{\delta} = 0.5(\boldsymbol{1}/\boldsymbol{\pi} - \boldsymbol{e}^2)$ ▷ second-order error
$\boldsymbol{a} = \phi'(\boldsymbol{t}) \circ (\boldsymbol{W}^T(\boldsymbol{\pi} \circ \boldsymbol{e}) + \boldsymbol{A}^T\boldsymbol{\delta})$ ▷ Total propagated error Eq. 3
$\boldsymbol{t} \leftarrow \boldsymbol{t} + \tau^{-1}(-\boldsymbol{t} + \boldsymbol{a})$ ▷ Neuronal dynamics Eq. 2 without top down influence
inferred_labels[$\boldsymbol{d}$] = argmax($\boldsymbol{t}$) ▷ Most probable class index

---

8. Mapping to cortical circuits

Here we present an overview of our proposed mapping to cortical circuits.

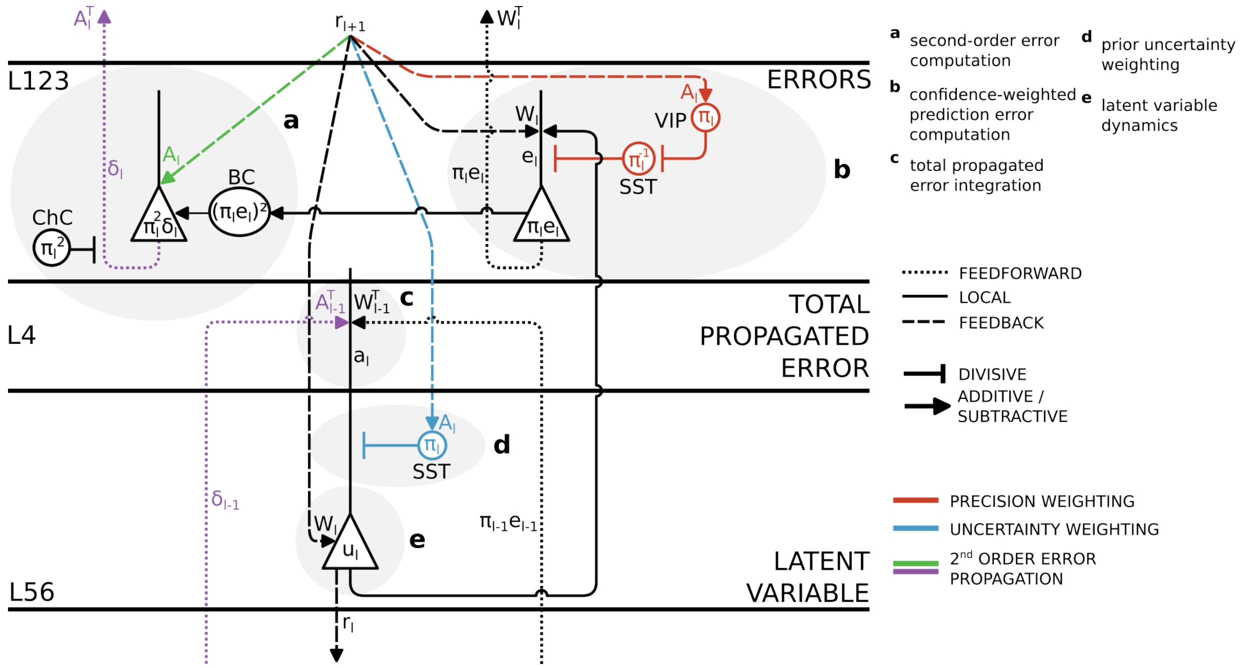

Fig. S3. Confidence estimation in cortical circuits. Cortical circuit for neuronal dynamics of inference (as described in Eq. 2 [$\tau\boldsymbol{u}_l = -\boldsymbol{u}_l + \boldsymbol{\mu}_l + \boldsymbol{\pi}_l^{-1} \circ \boldsymbol{a}_l$] and Eq. 3 [$\boldsymbol{a}_l = \boldsymbol{r}_l' \circ (\boldsymbol{W}_{l-1}^T(\boldsymbol{\pi}_{l-1} \circ \boldsymbol{e}_{l-1}) + \boldsymbol{A}_{l-1}^T\boldsymbol{\delta}_{l-1})$]). Representations [$\boldsymbol{u}_l$] are held in the somatic membrane potential of L6p. Top-down synapses carrying predictions [$\boldsymbol{\mu}_l = \boldsymbol{W}_l\boldsymbol{r}_{l+1}$] directly excite L6p at proximal dendrites (e). Bottom-up confidence-weighted prediction errors [$\boldsymbol{W}_{l-1}^T(\boldsymbol{\pi}_{l-1} \circ \boldsymbol{e}_{l-1})$] and second-order errors [$\boldsymbol{A}_{l-1}^T\boldsymbol{\delta}_{l-1}$] are integrated into total error [$\boldsymbol{a}_l$] in the distal dendrites of L6p as described in Eq. 3 (c). This total error is then weighted by the prior uncertainty [$\boldsymbol{\pi}_l^{-1}$] through divisive dendritic inhibition realized by infragranular SST-expressing interneurons (L56-SST) (d). Top-down predictions [$\boldsymbol{\mu}_l = \boldsymbol{W}_l\boldsymbol{r}_{l+1}$] and local representations [$\boldsymbol{u}_l$] are compared in dendrites of L3e. Confidence weighting is then realized through gain modulation of these dendrites by the disinhibitory VIP-expressing (VIP) and SST-expressing (L23-SST) interneurons motif (b). Here we propose a more speculative but detailed circuit for second-order errors computation. L3δ integrate top-down confidence estimates [$\boldsymbol{\pi}_l$] and local squared confidence-weighted prediction errors [$(\boldsymbol{\pi}_l \circ \boldsymbol{e}_l)^2$] encoded in basket cells (BC) into re-weighted second-order errors [$\boldsymbol{\pi}_l - (\boldsymbol{\pi}_l \circ \boldsymbol{e}_l)^2 = \boldsymbol{\pi}_l^2 \circ \boldsymbol{\delta}_l$] (a). Second-order errors [$\boldsymbol{\delta}_l$] are then sent up using the modulatory influence of chandelier cells (ChC) on the axonal initial segment of L3δ.

9. Predicted shapes of neural responses during experiments

In the following figure we present the shapes of neural responses predicted by our theoretical derivation in three simple experimental settings:

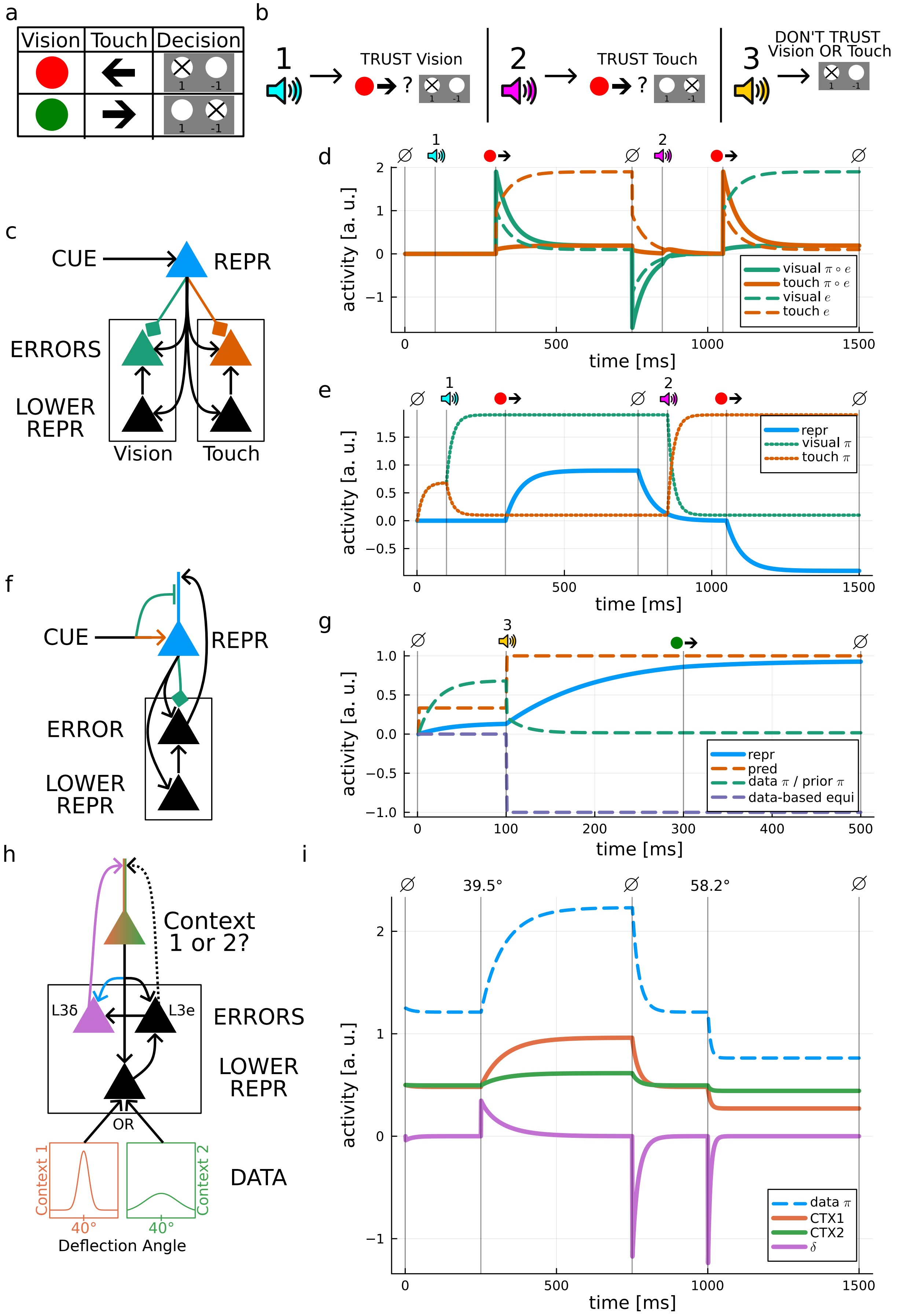

a
Vision
Touch
Decision
b
1
TRUST Vision
2
TRUST Touch
3
DON'T TRUST Vision OR Touch
c
CUE
REPR
ERRORS
LOWER REPR
Vision
Touch
d
activity [a. u.]
time [ms]
visual $\pi \circ e$
touch $\pi \circ e$
visual e
touch e
e
repr
visual π
touch π
f
CUE
REPR
ERROR
LOWER REPR
g
repr
pred
data π / prior π
data-based equi
h
Context 1 or 2?
L3δ
L3e
ERRORS
LOWER REPR
OR
Context 1
Context 2
40°
Deflection Angle
DATA
i
39.5°
58.2°
data π
CTX1
CTX2
δ

Fig. S4. (Previous page.) Predicted shapes of neural responses during experiments. For all simulations presented in this figure, we simulate Eqs. 2 and 3 (sometimes only the relevant parts of these equations) using a forward Euler discretization scheme with dt=1ms and τ=0.01ms. (a) Experimental setup. Sensory inputs are presented in two modalities: vision and touch. When the visual stimulus is red and the touch is on the left, decision 1 should be made ; when the visual stimulus is green and the touch is on the right, decision -1 should be made. (b) Experimental setup. Different auditory cues carry different meanings. Auditory cue 1 indicates that the vision modality should be trusted. If contradictory stimuli are presented, as here a red visual stimulus and a right touch, the decision should be the one associated with the visual stimulus: decision 1 (see a). Conversely, if auditory cue 2 is presented, the decision should be the one associated with the touch stimulus. Finally, if auditory cue 3 is presented, neither the visual nor touch stimuli should be trusted, and decision 1 should be taken every time. (c) A circuit representation of visual and touch confidence-weighted prediction errors computation. REPR refers to the representation neuron. (d) In this simulation, the agent is presented with auditory cues, followed 200ms later by a pair of stimuli (visual and touch) for an additional 450ms. This constitutes a trial, and there is a 100ms break between trials. Confidence-weighted prediction errors for the vision and touch modalities are depicted in green and orange plain lines respectively, while the raw errors are depicted in the same colors in dashed lines. (e) This is the same procedure as in d. Here, we additionally plot the decision variable, resulting from the activity in the representation neuron, in a blue plain line and the confidence estimates for the vision and touch modalities in green and orange dotted lines respectively. (f) A circuit representation for the integration of prior and data errors, with the divisive weighting of data errors by prior confidence. (g) In this simulation, the agent is presented with an auditory cue, followed 200ms later by a pair of stimuli (visual and touch) for an additional 200ms. The decision variable is depicted in a blue plain line, the contextual prediction by a dashed orange line, the ratio of data confidence over prior confidence by a green dashed line, and the equilibrium that would result from inference based only on the data, ignoring the prior, in a purple dashed line. (h) A circuit representation of second-order error computation. In this experiment, input (sensory) data to lower representation neurons can come from two different distributions with the same mean but different variances. The first distribution (Context 1, orange) has a low variance, while the second distribution (Context 2, green) has a high variance. (i) In this simulation, the agent is presented with stimuli (e.g. whisker deflections at different angles) for 500ms each with 250ms breaks between the stimuli presentations. Stimuli starts at 250ms and 1s. The second-order error is depicted in a purple plain line, the 2d context in orange and green lines, and the data confidence in a dashed blue line.

1. An experiment where an auditory cue indicates which of two sensory modalities should be trusted to make a decision (see Fig. S4a-e). Fig. S4d illustrates that the magnitude of confidence-weighted prediction errors (L3e somatic activity) for the modality that should be prioritized (attended to, that gives relevant information) is higher than for the ignored modality. Additionally, Fig. S4d illustrates that minimizing raw errors for the modality that has to be ignored is not a priority for the dynamics. Fig. S4e shows that the decision variable is indeed influenced mainly by the trusted modality, as indicated by the auditory cue.

2. An extension of the previous setting where an additional auditory cue can indicate directly what the decision should be (contextual prediction) and that sensory inputs should be ignored for this trial (see Fig. S4abfg). Fig. S4g indicates that in the context of this auditory cue, sensory inputs are successfully ignored and the decision variable tends towards the contextual prediction.

3. An experiment where the subject can receive sensory inputs (e.g. whisker deflections) with values (here, deflection angles) sampled from two different distributions with the same mean but different variances. The goal of the subject is to infer which of these two distributions it is receiving inputs from (see Fig. S4hi). In simulations depicted in Fig. S4i, the agent first observes a sensory input close to the mean, which makes it more likely that the input comes from the distribution with low variance. This produces a positive second-order error (δ): the observed deviation to the mean is smaller than expected. This correspondingly drives inference of the context (orange over green). Conversely, when the agent subsequently gets presented with a stimulus that is far away from the mean, negative second-order errors are produced and the context inference adjusts towards the distribution with high variance (green over orange).

References

1. B Millidge, A Seth, CL Buckley, Predictive coding: a theoretical and experimental review. *arXiv preprint arXiv:2107.12979* (2021).
2. A Ofner, RK Ratul, S Ghosh, S Stober, Predictive coding, precision and natural gradients. *arXiv preprint arXiv:2111.06942* (2021).
